# Glial Cell Line-derived Neurotrophic Factor and Retinoic Acid Synergy Unlocks Neurogenesis in Adult Myenteric Glia/Neural Progenitors

**DOI:** 10.1016/j.jcmgh.2025.101722

**Published:** 2026-01-05

**Authors:** Christopher Y. Han, Vipin Chauhan, Jessica L. Mueller, Aki Kashiwagi, Alan J. Burns, Rhian Stavely, Abigail R. Leavitt, Harsh Panchal, Takahiro Ohkura, Leah C. Ott, Ahmed A. Rahman, Ryo Hotta

**Affiliations:** Department of Pediatric Surgery, Massachusetts General Hospital, Harvard Medical School, Boston, Massachusetts

The culture of myenteric enteric neural stem/progenitor cells (ENPs) is: (1) an important resource for understanding the biology of enteric neurons (ENs) and enteric glial cells (EGCs)^1^, ^2^, ^3^, ^4^; and (2) a promising cell therapy for regenerating the enteric nervous system (ENS) in the gastrointestinal tract of patients with enteric neuropathies.^5^^,^^6^ Despite this growing interest in modeling the ENS in vitro, current protocols remain suboptimal, with postnatal-derived cell cultures often dominated by EGCs and enteric mesenchymal cells (EMCs) at the expense of ENs.^7^, ^8^, ^9^ Understanding the factors controlling culture heterogeneity and neurogenesis is essential for optimizing in vitro protocols and developing restorative cell therapies. Here, we investigated variables including donor age, culture adherence, and growth factors to understand and leverage their influence on the cellular composition of postnatal ENS cultures, with the aim of enhancing postnatal neurogenesis.

To determine the impact of donor age on the properties of ENS culture, neurospheres were cultured^1^ from the muscularis propria of 0.5- and 12-month-old BAF53b::TdTomato (TdT); Plp1-GFP dual-reporter mice^2^ to selectively label enteric glial/neural progenitor cells (EG/NP) (GFP fluorescence) and ENs (TdT fluorescence) (Figure 1*A*). *Sox10* (EG/NP) expression was unchanged between 0.5- and 12-month neurospheres, whereas *Elavl4* (EN) decreased and *Pdgfra* (EMC) increased with age (Figure 1*Ai*). In neurosphere image analysis, BAF53b-TdT fluorescence showed that neurogenesis was reduced by 2 months, with further decline at 12 months (Figure 1*Aii*, Supplementary Figure 1*A* and *B*). Postnatal EG/NPs acutely lose their neuronal differentiation potential similar to in vivo observations,^10^ emphasizing age as a key experimental and translational variable for the production of ENs.

**Figure 1:**
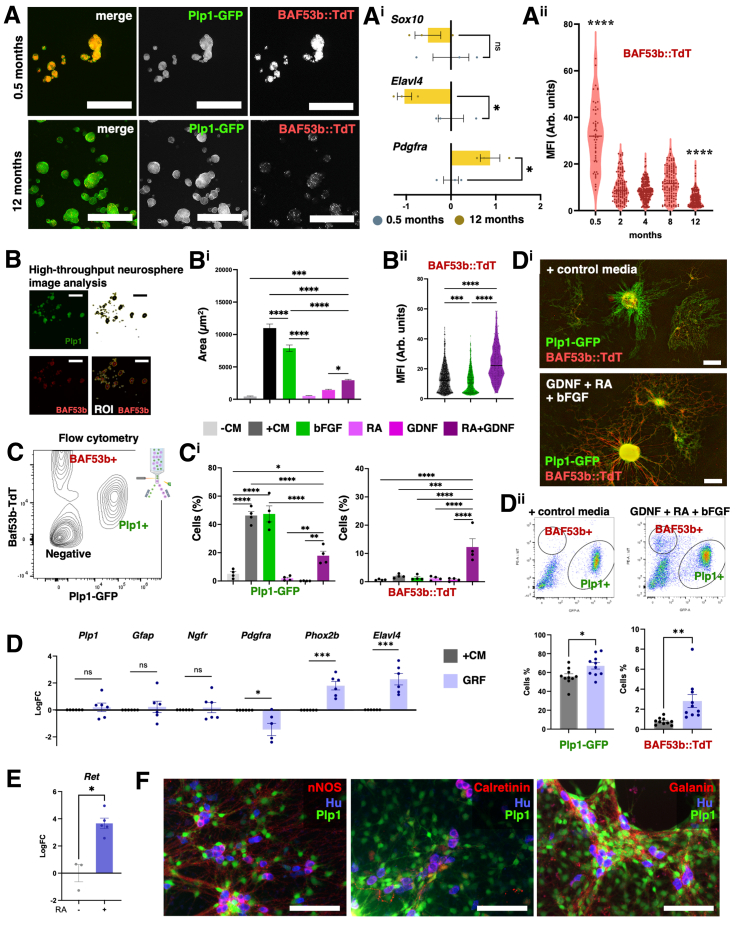
**(*A*) Neurospheres from 0.5- and 12-month-old BAF53b::TdT;Plp1-EGFP mice.** (*Ai*) PCR data from these neurospheres. n = 3 mice per group. (*Aii*) Quantification of MFI of TdT in GFP-binarized ROI. n = 51–224 neurospheres per group; individual data points from all wells combined for each age group. (*B*) Neurosphere analysis method of GFP signal binarization, region of interest (ROI) selection, and TdT mean fluorescence intensity (MFI) quantification. (*Bi*) Area (μm^2^) per well covered by neurospheres in −CM, +CM, bFGF, GDNF, RA, and RA+GDNF media; n = 4 cultures. (*Bii*) TdT MFI in GFP-defined neurospheres. n = 248–639 neurospheres. (*C*) Flow cytometry gating examples. (*Ci*) Percentage of EG/NPs (Plp1-EGFP) and ENs (BAF53b-TdT). n = 4 cultures. (*D*) Quantitative PCR in neurospheres from the +CM and GRF media groups. n = 6 independent cultures per group. (*Di*) Representative images of monolayer cultures in +CM and GRF media. (*Dii*) Representative flow cytometry plots and quantification of EG/NPs and ENs in the +CM and GRF media groups. n = 10 independent cultures per group. (*E*) Expression of *Ret* in neurospheres cultured with and without RA. n = 3–5 cultures. (*F*) nNOS, Calretinin, and Galanin neurons in GRF media cultures. All data are mean ± SEM unless stated. Unpaired *t*-test (*Ai, Di, Dii, E*), Kruskal-Wallis ANOVA with Dunn’s posthoc test (*Aii*), One-way ANOVA with Holm-Sidak (*Bi, Bii, Ci*), ∗*P* < .05; ∗∗*P* < .01; ∗∗∗*P* < .001; ∗∗∗∗*P* < .0001. Scale bars = 1000 um (*A*), 500 um (*D*), 100 um (*F*).

To test the influence of culture adherence conditions, adult-derived neurospheres were grown in free-floating 3D cultures and in adherent monolayers (Supplementary Figure 1*C* and *D*). *Plp1* and *Ngfr* levels were similar in both conditions, whereas cells were less differentiated in monolayers with reduced *Gfap, Phox2b,* and *Elavl4* (Supplementary Figure 1*E*). Neurospheres in 3D cultures yielded fewer singlets by flow cytometry after digestion than monolayers (26.7% ± 1.7% vs 32.3% ± 0.44%; *P* < .01), consistent with neurospheres having greater resistance to dissociation. Thus, monolayer cultures, which are more amenable to dissociation, yielded better recovery of ENs and EG/NP singlets than free-floating cultures (Supplementary Figure 1*F*). Monolayer cultures therefore offer advantages for protocols requiring cell suspensions, whereas 3D conditions improve cell differentiation (Supplementary Figure 2*A*).

To manipulate and optimize adult EG/NP proliferation and neurogenesis the ligands basic fibroblast growth factor (bFGF), retinoic acid (RA), and glial cell line-derived neurotrophic factor (GDNF), which play key roles in neural development, were tested individually and in combination (Supplementary Figure 3*A*). Positive control media (+CM), known to support neurosphere formation,^1^ and basal control media (−CM) were included as comparators (Supplementary Figure 2*B*). bFGF expanded adult progenitors but did not increase numbers of ENs (Figure 1*B* and *Ci*). RA + GDNF synergistically increased neuronal differentiation 6.2-fold while moderately expanding EG/NPs (Figure 1*B* and *Ci*). To combine the proliferative and neurogenic properties of these growth factors, we utilized GRF media (GDNF + RA + bFGF) and compared it with +CM (Figure 1*D* and *Dii*, Supplementary Figure 3*B*). After 2 weeks, *Plp1, Gfap*, and *Ngfr* were comparable between groups, whereas *Pdgfra* was lower and *Phox2b/Elavl4* were higher in GRF (Figure 1*D*). Flow cytometry after monolayer expansion (Figure 1*Di*) showed GRF increased ENs 3.6-fold and EG/NPs 1.2-fold (Figure 1*Dii*), indicating enhanced neurogenesis without compromising progenitor maintenance. GRF did not significantly increase survival of sorted mature ENs as compared with +CM, indicating GRF enhances EN numbers via neurogenesis (Supplementary Figure 3*C–E*). In sorted Plp1+ EG/NPs, RA promoted the expression of *Ret* >12-fold, providing a mechanism for RA to enhance GDNF-driven neurogenesis via its receptor (Figure 1*E*). Purified EG/NPs in GRF media produced ENs, including representative intrinsic ENS subtypes nNOS, Calretinin, and Galanin (Supplementary Figure 4*A*; Figure 1*F*).

To translate our findings with mouse cells to human, we obtained human muscularis propria from patients aged 9 weeks to 49 years (Supplementary Figure 2*C*; Figure 2*A* and *B*), generated neurospheres, and cultured them in +CM or GRF. After 2 weeks, GRF neurospheres expressed increased *PLP1*, *NGFR*, and *PHOX2B* compared with +CM (Figure 2*C* and *D*). Similar to rodents, an age-related decline in *PHOX2B*, but not *PLP1*, was observed (Supplementary Figure 4*B*). Following monolayer expansion (Figure 2*E*), GRF yielded fewer cells (Figure 2*F*), but higher proportions of ITGA6+, NGFR+, and TUBB3+ cells by immunostaining (Figure 2*G–I'*). Thus, like our observations with mouse cells, these data indicate that GRF media provides highly neurogenic human cell populations in vitro.

**Figure 2:**
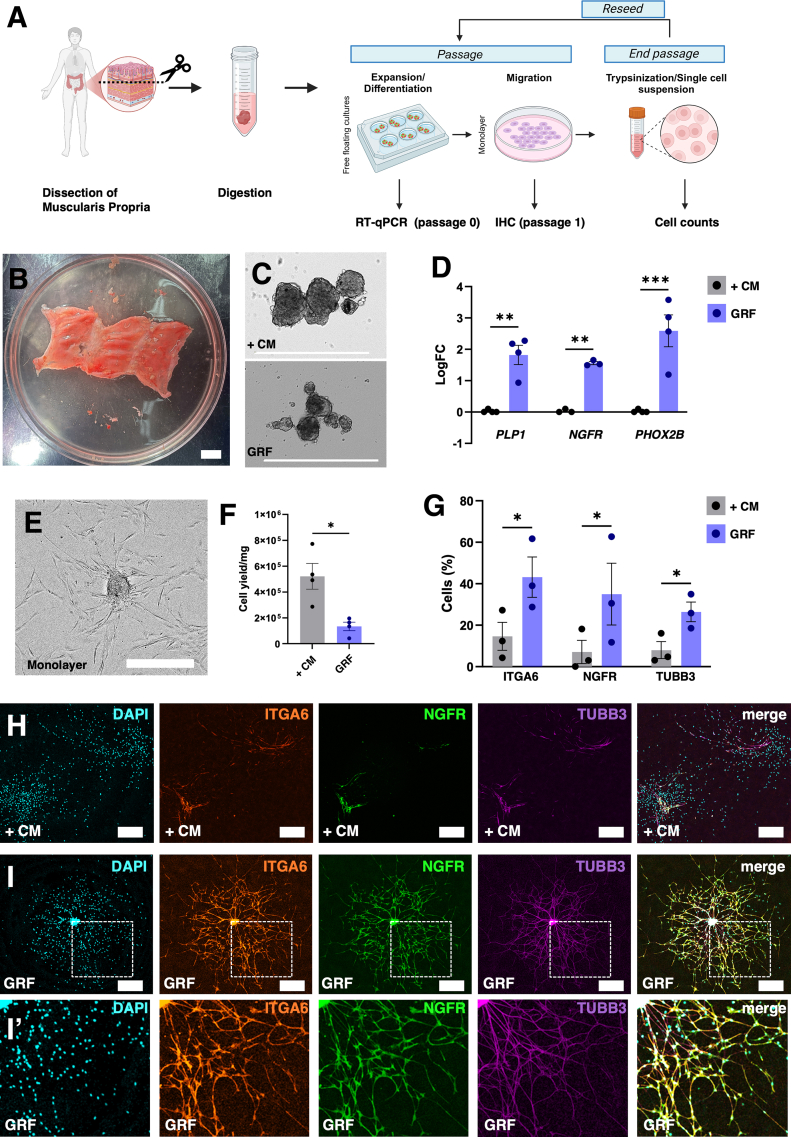
**(*A*) Schematic of human intestinal neurospheres cultured in +CM or GRF media.** (*B*) Representative muscularis propria tissue after dissection. (*C*) Representative neurospheres in +CM and GRF media. (*D*) EG/NP markers *PLP1, NGFR*, and neuronal marker *PHOX2B* after 10–14 days free-floating culture; n = 3–4 subjects. (*E*) Neurospheres transferred to monolayer in GRF media. (*F*) Cell yield per mg of tissue; n = 4. (*G*) Percentage of ITGA6+, NGFR+, and TUBB3+ cells; n = 3; 2-way ANOVA. (*H* and *I*) Representative immunohistochemistry images for ITGA6, NGFR, and TUBB3 in +CM (*H*) and GRF (*I*). All data are mean ± SEM. Two-way ANOVA with Holm-Sidak (*D* and *G*), ratio paired *t*-test (*F*). ∗*P* < .05; ∗∗*P* < .01; ∗∗∗*P* < .001. Scale bars: 1 cm (*B*), 500 μm (*C, E, H* and *I-I'*).

Our findings highlight that culture conditions can be tailored to dictate the numbers of glial and neuronal cells that can be generated in culture, depending on experimental or translational goals. bFGF robustly expanded adult EG/NPs but with limited production of neurons, while RA + GDNF increased neuronal differentiation, thereby demonstrating the ability to tune cell fate. Our results not only align with reports of age-related decline in ENS neurogenesis in vivo,^10^ but importantly, also demonstrate that adult EG/NPs retain neurogenic capability when appropriately stimulated in vitro. Insights from iPSC-derived systems could further refine approaches to culture adult progenitors, and future studies should assess potential sex-dependent effects. Optimizing culture conditions and exposure to pro-neurogenic cues represents a critical step towards the establishment of reproducible in vitro models of the ENS and the development of adult neural cell therapies, where precise manipulation of EG/NPs and ENs is essential.

## Supplementary Material

Supplementary Methods

Extended PDF
