# Supplementary material for: Glial Cell Line-derived Neurotrophic Factor and Retinoic Acid Synergy Unlocks Neurogenesis in Adult Myenteric Glia/Neural Progenitors
Source: Cell Mol Gastroenterol Hepatol. 2026 Jan 5;20(5):101722. doi: 10.1016/j.jcmgh.2025.101722 (PMC12933833; doi:10.1016/j.jcmgh.2025.101722)
Supplement: Supplementary Figures [file mmc1.pdf]

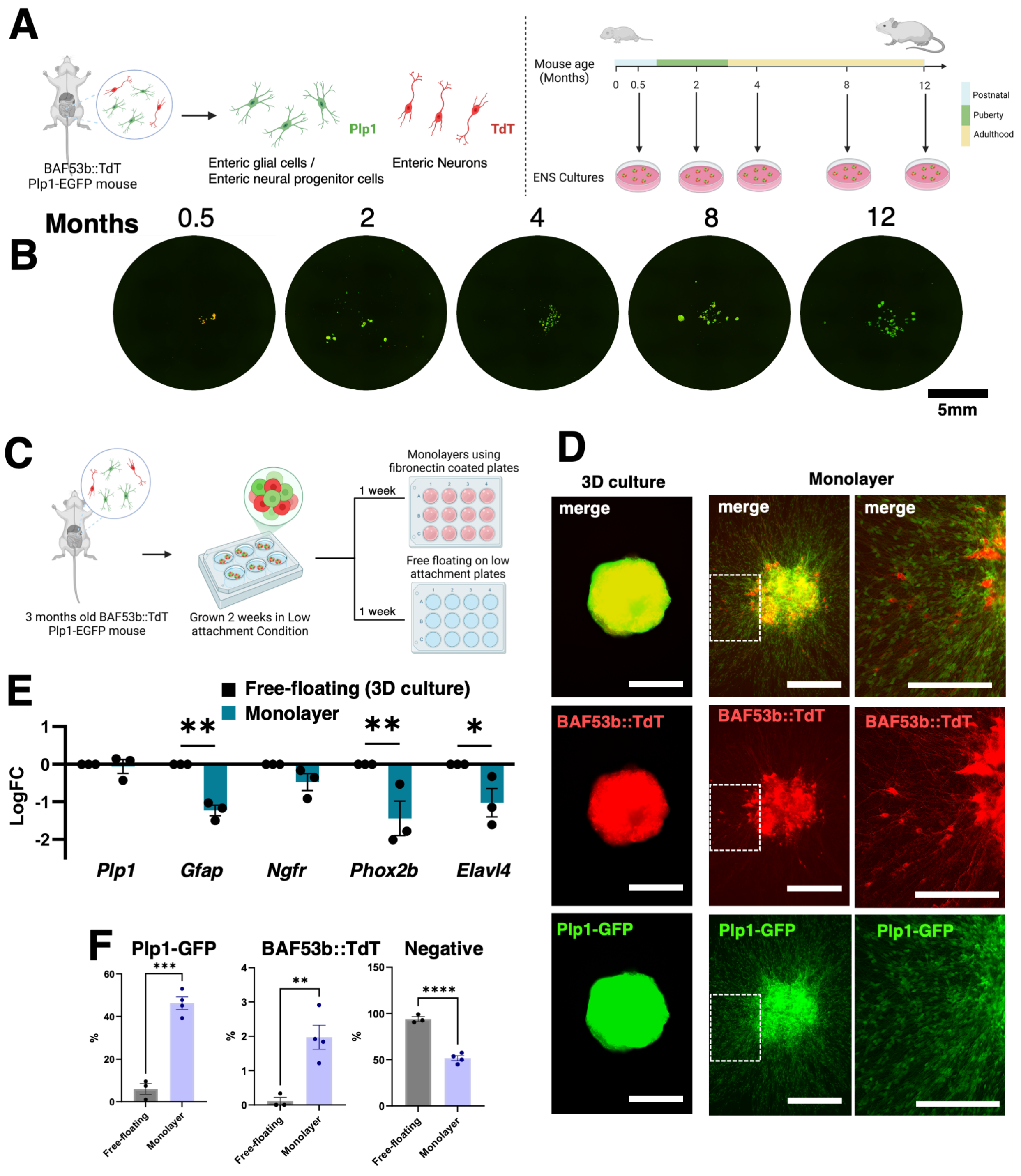

Supplementary Figure 1. A) Neurogenesis declines in enteric neurospheres with increasing age of mice. Schematic overview of neurosphere cultures from BAF53b::TdT; Plp1-EGFP dual-reporter mice at a range of ages. **B)** Cells isolated from BAF53b::TdT; Plp1-EGFP dual-reporter mice at 2 weeks, 2 months, 4 months, 8 months, and 1 year of age give rise to GFP+ and TdT+ neurospheres in culture, with Plp1-EGFP serving as a EG/NP marker and BAF53b::TdT as a neuronal marker. Images are representative of whole wells in culture plates. Scale bar = 5mm. **C)**

Culture format influences cell composition and assay sensitivity in enteric neural cultures. Schematic representation of neurosphere cultures grown under monolayer and free-floating conditions (3D culture) from BAF53b::TdT; Plp1-EGFP mice. **D)** Representative images of neurospheres generated from BAF53b::TdT; Plp1-EGFP dual-reporter mice, in the free-floating group at the 3-week timepoint of culture and the monolayer group at the 3-week timepoint of culture. Scale bars = 500um and 250um for right insets. **E)** Quantitative PCR of *Plp1, Gfap, Ngfr, Phox2b*, and *Elavl4* in neurospheres from the free-floating and monolayer groups. Data are shown as mean ± SEM. Two-way ANOVA with Holm-Sidak posthoc test, $^{*}p < 0.05$, $^{**}p < 0.01$; n = 3 mice per group. **F)** Quantification of flow cytometry for EG/NPs (Plp1-EGFP), ENs (BAF53b::TdT), and EMCs (double-negative cells) from free-floating and monolayer groups. Data are shown as mean ± SEM. One-way ANOVA with Holm-Sidak posthoc test, $^{**}p < 0.01$, $^{***}p < 0.001$, $^{****}p < 0.0001$; n = 3-4 wells per group.

A) Comparison between monolayer and neurosphere culture systems

Culture format	Benefits	Assays
**Free-floating neurospheres** (3D culture)	- Better mimics *in vivo* 3D structure<br>- Higher rates of neuronal differentiation<br>- More scope for expansion	- PCR<br>- Transgenic fluorescence<br>- Neurosphere coverage
**Monolayer**	- Easier to maintain and passage<br>- More amenable to generation of single-cell suspensions for controlled passaging and assays	- Immunocytochemistry<br>- Neurite outgrowth<br>- Cell yield<br>- Flow cytometry<br>- PCR

B) Media definitions

Media	+CM	-CM	FGF	RA	GDNF	GR	GRF
**DMEM**	+	+	+	+	+	+	+
**NeuroCult**	+	-	-	-	-	-	-
**B27**	+	+	+	+	+	+	+
**N2**	+	+	+	+	+	+	+
**Anti-Anti**	+	+	+	+	+	+	+
**2-ME**	+	-	-	-	-	-	-
**IGF**	+	-	-	-	-	-	-
**bFGF**	+	-	+	-	-	-	+
**RA**	+	-	-	+	-	+	+
**GDNF**	-	-	-	-	+	+	+

+CM, positive control media; -CM, negative control media; bFGF, fibroblast growth factor; RA, retinoic acid; GDNF, glial cell line-derived neurotrophic factor; GR, GDNF+RA; GFR, GDNF+RA+FGF; 2-ME, 2-mercaptoethanol; IGF, insulin-like growth factor, bFGF; Basic fibroblast growth factor.

C) Human sample details

ID	Age	Sex	Procedure	Tissue
**1**	9-week-old	M	Resection for Meckel's diverticulum	Ileum
**2**	6.5-month-old	M	Colostomy Closure for anorectal malformation	Sigmoid Colon
**3**	36-year-old	F	Right colectomy for colonic tumor	Transverse Colon
**4**	49-year-old	F	Colostomy closure following prior diversion for colorectal cancer	Transverse Colon

Supplementary Figure 2. A) Comparison of culture formats used in this study, outlining key benefits and downstream applications. Free-floating neurospheres (3D culture) better recapitulate *in vivo* three-dimensional architecture, exhibit higher rates of neuronal differentiation, and allow greater capacity for cellular expansion. Neurosphere cultures were used for gene expression analysis by PCR, assessment of transgenic reporter fluorescence, and quantification of neurosphere formation and coverage. Monolayer cultures are easier to maintain and passage and are more amenable to generation of single-cell suspensions for controlled passaging and downstream assays. Monolayer systems can be used for immunocytochemistry, neurite outgrowth analysis, cell yield quantification, flow cytometry, and PCR. **B)** Composition of culture media used across experimental conditions. Media components are indicated as present (+) or absent (−). +CM denotes positive control media and −CM denotes negative control media. Abbreviations: DMEM, Dulbecco's Modified Eagle Medium; bFGF/FGF, basic fibroblast growth factor; RA, retinoic acid; GDNF, glial cell line–derived neurotrophic factor; GR, GDNF + RA; GRF, GDNF + RA + FGF; B27 and N2, neuronal supplements; Anti-Anti, antibiotic–antimycotic; 2-ME, 2-mercaptoethanol; IGF, insulin-like growth factor. **C)** Clinical and demographic details of human intestinal samples used for primary cell isolation, including age, sex, surgical indication, and tissue source.

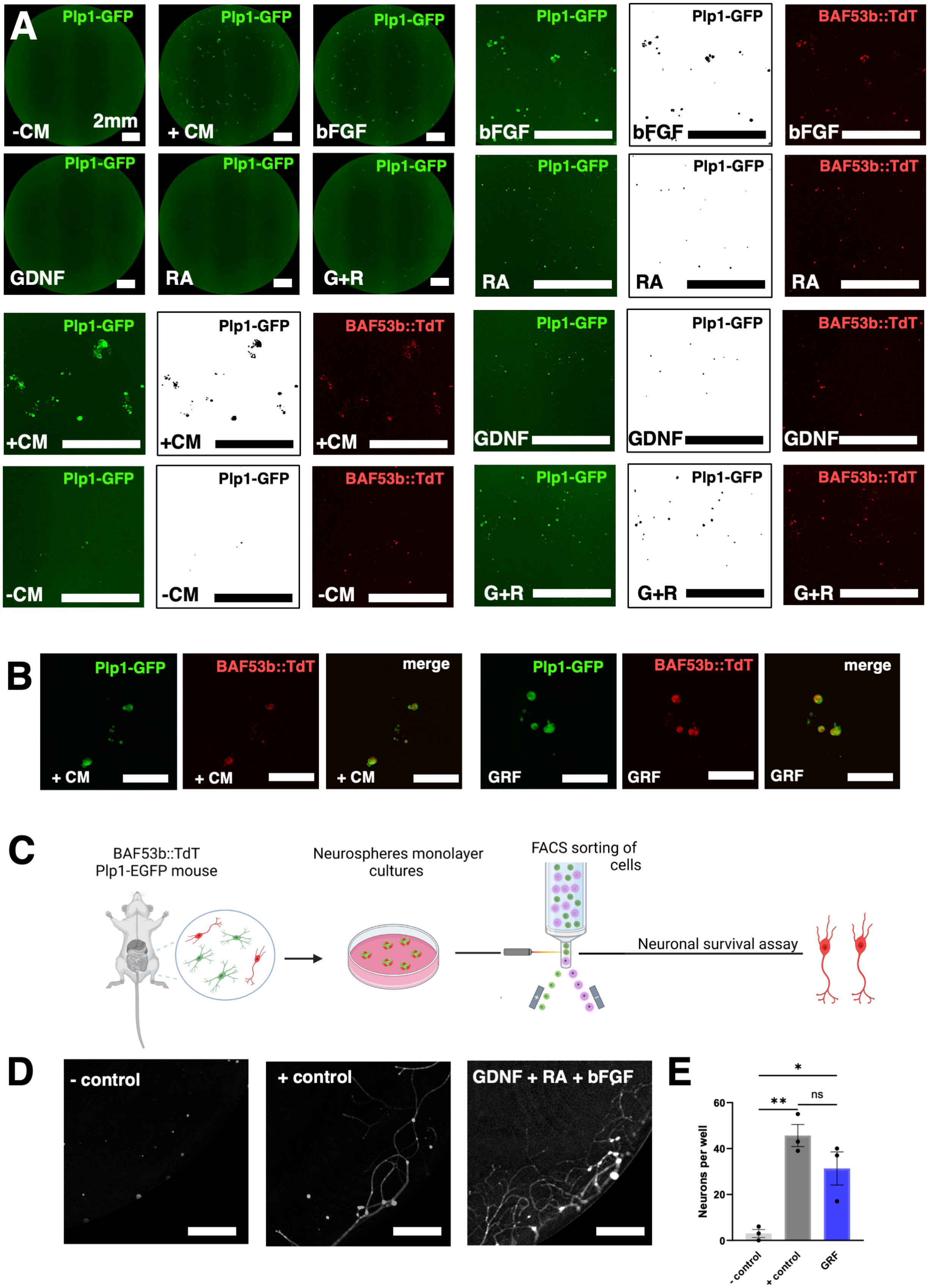

A
Plp1-GFP
BAF53b::TdT
-CM
+ CM
bFGF
GDNF
RA
G+R
2mm
B
merge
GRF
C
BAF53b::TdT
Plp1-EGFP mouse
Neurospheres monolayer cultures
FACS sorting of cells
Neuronal survival assay
D
- control
+ control
GDNF + RA + bFGF
E
Neurons per well
60
40
20
0
*
**
ns
- control
+ control
GRF

Supplementary Figure 3. A) Representative GFP-channel images of neurospheres generated from 3-month-old BAF53b::TdT; Plp1-EGFP dual-reporter mice and cultured in negative control media (-CM), positive control media (+CM), bFGF media, RA, GDNF or GDNF + RA (G+R) media conditions. Representation of GFP fluorescence binarization and TdT fluorescence for neurospheres. Scale bars = 2mm. **B)** GDNF, RA and bFGF (GRF) improves enteric neuronal composition while maintaining enteric glial/neural progenitor expansion in adult-derived neurosphere cultures. Representative images of neurospheres generated from BAF53b::TdT; Plp1-EGFP dual-reporter mice, cultured in the +CM media and GDNF + RA + bFGF (GRF) media. **C)** GDNF, RA and bFGF (GRF) improves enteric neuronal composition while maintaining enteric glial/neural progenitor expansion in adult-derived neurosphere cultures. Schematic representation of neurosphere cultures grown in positive control media (+CM), bFGF, and GDNF + RA + bFGF (GRF) media. **B)** Representative images of neurospheres generated from BAF53b::TdT; Plp1-EGFP dual-reporter mice, cultured in the +CM media or GDNF + RA + bFGF (GRF) media. Scale bars = 2mm. **C)** Schematic illustration of ENs (BAF53b::TdT) sorted from BAF53b::TdT; Plp1-EGFP dual-reporter mice, used for EN survival assays. **D)** Representative TdT-channel images of ENs (BAF53b::TdT) sorted from BAF53b::TdT; Plp1-EGFP dual-reporter mice, then cultured in negative control (-CM), positive control (+CM), or GDNF + RA + bFGF (GRF) media conditions, at the 2-week timepoint of culture. Scale bars = 500um. **E)** Quantification of neurons per well in ENs (BAF53b::TdT) as above. Data are shown as mean ± SEM. One-way ANOVA with Holm-Sidak posthoc test, $^{*}p < 0.05$, $^{**}p < 0.01$; $n = 3$ independent cultures per group.

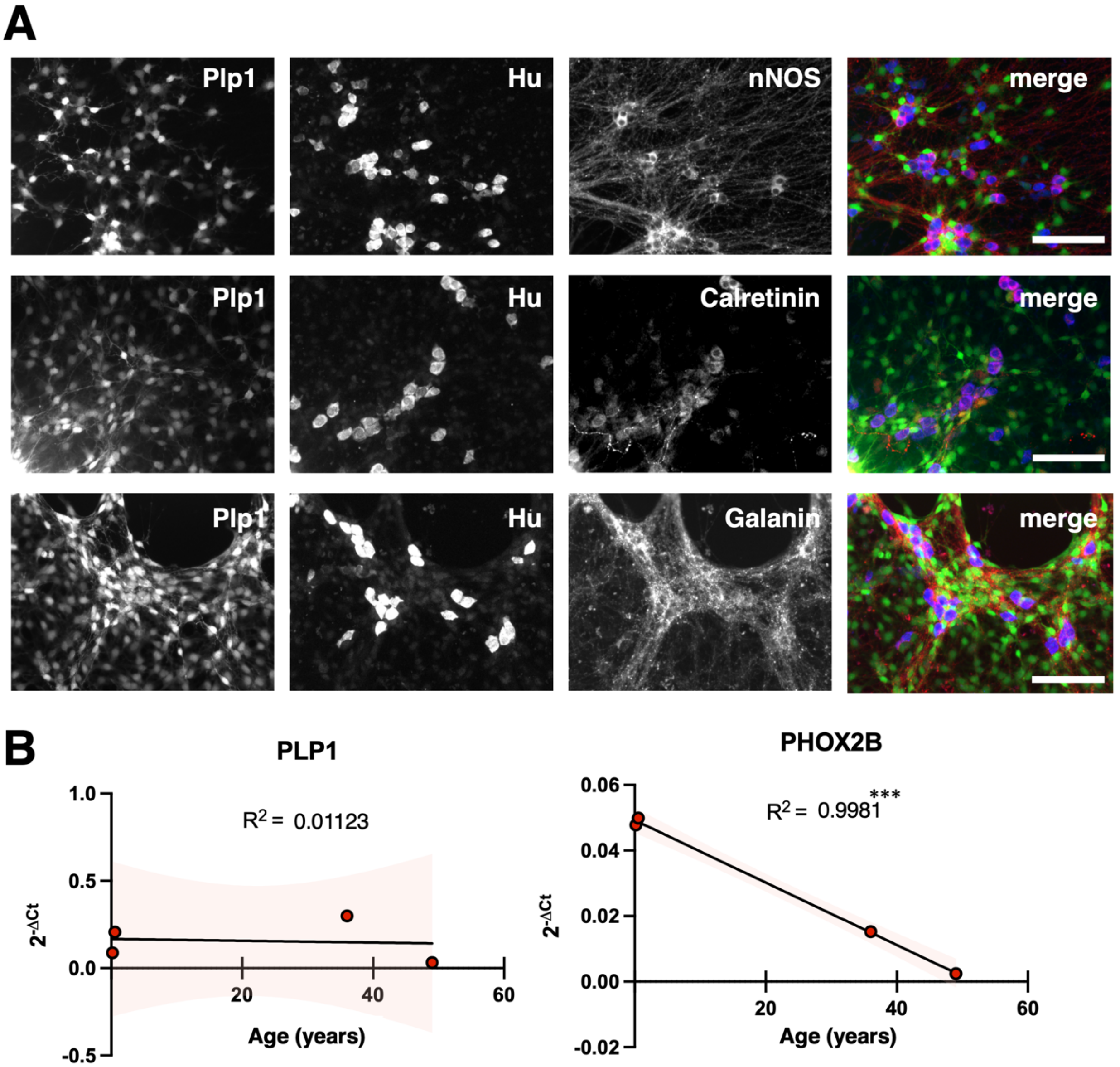

Supplementary Figure 4. A) Enteric glial/neural progenitor cells (EG/NPs) sorted for Plp1-GFP expression and cultured in GDNF + RA + bFGF (GRF) media undergo neurogenesis and give rise to nNOS, Calretinin and Galanin immunoreactive neurons in culture. Scale bars = 100 um. **B)** Linear regression analysis of the expression of PLP1 or PHOX2B ($2^{-\Delta Ct}$) against the age (years) of human subjects from which cultures were derived. Data are from samples grown in GRF media. Data shows individual points (red circles), regression line (black line) and 95% confidence intervals (shaded region). Significance of the slope, $^{***}p < 0.001$.
