# Supplementary material for: Glial Cell Line-derived Neurotrophic Factor and Retinoic Acid Synergy Unlocks Neurogenesis in Adult Myenteric Glia/Neural Progenitors
Source: Cell Mol Gastroenterol Hepatol. 2026 Jan 5;20(5):101722. doi: 10.1016/j.jcmgh.2025.101722 (PMC12933833; doi:10.1016/j.jcmgh.2025.101722)
Supplement: Supplementary Methods [file mmc2.pdf]

Materials/Methods

All authors had access to the study data and had reviewed and approved the final manuscript. All data and analytic methods are provided in the manuscript. All raw data and study materials will be made available to other researchers upon reasonable request.

Animals

Animal experimentation was performed according to experimental protocols approved by the Institutional Animal Care and Use Committees IACUC (2009N000239) of Massachusetts General Hospital. Plp1-EGFP mice were gifted to the Goldstein laboratory by Wendy Macklin [1]. BAF53b::Cre (stock number 027826) and (R)26-tdTomato (stock number 007914) mice were purchased from The Jackson Laboratory. BAF53b::tdT; Plp1-EGFP dual glia and neuron reporter mice were generated as previously described [2-4]. All mice were housed and bred under specific-pathogen-free conditions at the Center for Comparative Medicine animal facility at Massachusetts General Hospital (MGH). Rodents were housed in Allentown Inc. rectangular caging (160 cages per individually ventilated cage racks; which uses blower at 60 air changes per hour) under a 12h:12h light:dark cycle from 7 am – 7 pm. Bedding consisted of Hardwood Sanichip; with Carefresh nesting material and mice had access to Prolab Isopro RMH 3000 chow mix (ScottPharma) *ad libitum*.

Generation of Heterogenous Mouse Cultures and Study Designs

Mice were euthanized and the small intestine was removed from duodenum to terminal ileum. The smooth muscle-myenteric plexus (SMMP) layer was dissected from underlying tissue in PBS and digested for 35 minutes at 37 °C in dispase (250 µg/mL; #07923 STEMCELL Technologies,

Vancouver, BC) and collagenase XI (#C7657-25MG 1 mg/mL; Sigma–Aldrich, Darmstadt, Germany). Following digestion, the cells were filtered through a 40-µm filter and plated in their respective media formulation **(Supplementary Figure 2B)**. Positive control media (+CM) contained a 1:1 mixture of DMEM (+ Glucose + Glutamine, Thermo Fisher, Waltham, MA) and NeuroCult Basal Media (#05700, STEMCELL Technologies,) supplemented with 20 ng/mL bFGF (STEMCELL Technologies, #78003), 20 ng/mL IGF-I (#RP-10931 Thermo Fisher, Waltham, MA)1, 2% B-27 supplement (#, 12587001, Gibco Thermo Fisher), 1% N-2 supplement (#17502001, Gibco Thermo Fisher), 50 mM 2-mercaptoethanol(#21985023, Thermo Fisher), 75 ng/mL all-trans retinoic acid (#R2625-100MG, Sigma-Aldrich) and 1% Antibiotic-Antimycotic (#15240062, Gibco Thermo Fisher) as previously reported [5]. Negative control media (-CM) contained only 2% B27 supplement, 1% N_2 supplement and 1% Antibiotic-Antimycotic in DMEM (#11995073, Gibco Thermo Fisher) and was supplemented with different combinations of bFGF (20 ng/mL), all-trans retinoic acid (75 ng/mL) and/or GDNF (50ng/mL) (#10788-108, FUJIFILM Irvine Scientific, Santa Ana, CA). All experiments were conducted using a standard humidified cell culture incubator (37˚C, 5% CO_2 and atmospheric O_2) with media supplemented once weekly.

High throughput image-based analysis of BAF53b::tdT; Plp1-EGFP neurospheres.

For experiments examining the effects of age and media compositions on heterogenous cell cultures, BAF53b::tdT; Plp1-EGFP mice were sacrificed and single cell suspensions were generated as above. Cells were plated at a density of 50,000 cells per well in 24-well ultra-low attachment plates (Corning Costar® 24-well Clear Flat Bottom Ultra-Low Attachment Multiple Well Plates Cat# 3473). After two weeks, whole-plate tile scans were acquired using a Keyence BZX-700 All-In-One Microscopy System (Keyence America, Itasca). Image processing was

performed in ImageJ (NIH, Bethesda), where individual wells were isolated and analyzed as follows: The Plp1-EGFP channel was binarized using auto-thresholding with the MaxEntropy algorithm, followed by despeckling to remove outliers smaller than 2 μm. Neurospheres were segmented using the Watershed function, and the "Analyze Particles" command was used to quantify and measure the area occupied by Plp1-EGFP+ neurospheres, filtering for objects >20 μm. Regions of interest (ROIs) for each neurosphere generated from the particle analysis were utilized to measure the mean grey value (mean fluorescence intensity, MFI) of BAF53b::tdT expression within each neurosphere.

RNA Isolation / qPCR

RNA isolation and RT-qPCR was performed as previously described [2, 6] via a RNeasy Mini kit (#74106, Qiagen, Hilden, Germany) as of manufacturer's instructions. Primer sequences for gene amplification in mouse and human samples are:

Gene	Forward sequence (5'→3')	Reverse sequence (5'→3')
**Gapdh (Ms)**	AGG TCG GTG TGA ACG GAT TTG	TGT AGA CCA TGT AGT TGA GGT CA
**Plp1 (Ms)**	TGA GCG CAA CGG TAA CAG G	GGG AGA ACA CCA TAC ATT CTG G
**Gfap (Ms)**	GGG GCA AAA GCA CCA AAG AAG	GGG ACA ACT TGT ATT GTG AGC C
**Ngfr (Ms)**	CCT GGA CAG TGT TAC GTT CTC	ACA CAG GGA GCG GAC ATA CT
**Phox2b (Ms)**	GGG CTA AGT TTC GCA AGC AG	CAG TGC TGT CGG GAT CAG TG
**Elavl4 (Ms)**	GCC TCA GGT GTC AAA TGG ACC	ACC CTA AAC TCT GTC CTG TGA T
**Pdgfra (Ms)**	ATG AGA GTG AGA TCG AAG GCA	CGG CAA GGT ATG ATG GCA GAG
**Sox10 (Ms)**	CGG ACG ATG ACA AGT TCC CC	GTG AGG GTA CTG GTC GGC T
**Ret (Ms)**	GCA TGT CAG ACC CGA ACT CC	CGC TGA GGG TGA AAC CAT CC
**GAPDH (Hu)**	GGA GCG AGA TCC CTC CAA AAT	GGC TGT TGT CAT ACT TCT CAT GG
**PLP1 (Hu)**	TGC TGA TGC CAG AAT GTA TGG	GCA GAT GGA CAG AAG GTT GGA
**NGFR (Hu)**	CCG TTG GAT TAC ACG GTC CAC	TGA AGG CTA TGT AGG CCA CAA
**TUBB3 (Hu)**	GGC CAA GGG TCA CTA CAC G	GCA GTC GCA GTT TTC ACA CTC

Quantification cycle (Ct) values were normalized to *Gapdh/GAPDH* expression within each sample as an internal control, and the Log2 fold change (FC) was calculated between

corresponding sample conditions from the same mouse or subject. All reactions were performed in duplicate.

Flow cytometry analysis

Analysis by flow cytometry was conducted on samples generated from BAF53b::tdT; Plp1-EGFP mice. To generate single cell suspensions, free-floating neurospheres were dissociated for 45 minutes using Accutase (STEMCELL Technologies), or monolayer cultures on fibronectin-coated plates (sigma, #F1141, 1:100 for 1h) were dissociated for 10 minutes into a single-cell suspensions using .025% Trypsin-EDTA (#25200056, Gibco Thermo Fisher) at 37C°C. Samples were filtered through a 35-µm cell strainer (#6475025, Electron Microscopy Sciences, Hatfield, PA) and stained with DAPI(#D1306, Invitrogen Thermo Fisher) serving as a viability marker. Cell sorting was performed with BD FACSAria cell sorter (BD Biosciences, Franklin Lakes, New Jersey) instruments. Flow cytometric analysis was conducted using FlowJo software (FlowJo, LLC, OR).

Neuronal survival assay

Cells were isolated from the small intestine of BAF53b::tdT; Plp1-EGFP mice as described above. ENs were isolated using fluorescence activated cell sorting (FACS) as previously described [4]. Briefly, the heterogenous population of cells was plated at a density of 5×10^4 cells/cm^2 on a fibronectin-coated (sigma, #F1141, 1:100 for 1h) 24-well cell culture plate cultured for 2 days in media comprised of DMEM/F12 media (ThermoFisher, Gibco) containing 10% FBS (#10438-018, ThermoFisher, Gibco) and 1% penicillin-streptomycin (ThermoFisher, Gibco, 15140122). Monolayers were trypinized as above and FACS was conducted using a BD FACSAria cell sorter (BD Biosciences) to collect GFP+ EG/NPs and tdT+ ENs.. For neuronal survival assays

BAF53b::tdT+ ENs were cultured in their respective media on 96-well fibronectin-coated flat-bottom plate at a density of 500 neurons per well. After two weeks in culture images of the entire wells were taken on a Keyence BZX-700 All-In-One Microscopy System and the number of neurons were quantified per well manually with ImageJ. Media was supplemented once weekly.

Immunocytochemistry

Monolayer cultures were fixed and immunolabeled as previously described [2, 7]. The following primary antibodies were utilized: rabbit anti-nNOS(C7D7) (4231, 1:200, Cell Signaling technology, Danvers, MA); rabbit anti-calretinin, (18-0211, 1:200, Invitrogen, Carlsbad, CA) rabbit anti-galanin (T4333, 1:2000, BMA Biomedicals, Augst, Switzerland), donkey anti-rabbit 647 (Invitrogen), mouse anti-tubulin β3 (801210, 1:400, conjugated to Alexa Fluor 647, BioLegend, San Diego, CA); mouse anti-CD271 (NGFR) (345104, 1:200, conjugated to FITC, Biolegend); rat anti-CD49f/ITGA6 (313616, 1:200; conjugated to APC, Biolegend). Cell nuclei were stained with DAPI (Invitrogen).

Human Gut Tissues

Colonic or ileal samples resected as part of required patient care at Massachusetts General Hospital were collected from subjects between 2 months and 49 years old **(Table 3)**. Tissues were stored overnight at 4°C in sterile PBS. The muscularis propria was mechanically separated from the mucosal, submucosal, and serosal layers using fine forceps and microdissection scissors under a stereoscopic microscope. For enzymatic dissociation, tissues were minced into approximately 5 mm fragments using sterile microdissection scissors and digested in a prewarmed enzymatic solution consisting of collagenase type XI (1 mg/mL) and dispase (0.6 U/mL) in DMEM/F12. The

digestion was carried out at 37°C for 4 h in a Mini Incubated Shaker (#76407-108, VWR International, Portland, OR) with intermittent trituration using glass serological pipettes of progressively smaller bore diameters (3 mm, 2 mm, and 1 mm) until the sample became liquefied. Large undigested tissue fragments were removed using a 1000 μm cell strainer (#4355100003 pluriStrainer, pluriSelect USA, El Cajon, USA), and single cells were obtained by passing the suspension through a 70 μm strainer (#22-363-548 Fisher Scientific, Pittsburgh, PA). Red blood cells were lysed by incubating the cell suspension in ACK Lysing Buffer (#A1049201, Gibco, ThermoFisher Scientific) for 7 min at room temperature.

Following isolation, cells were divided into equal aliquots and plated in their respective culture media at a density of 5–20 × 10^3 cells/cm^2 in ultra-low attachment plates for direct comparisons. As a positive control media (+CM) we utilized a defined formulation [8] which consisted of DMEM/F12(#11320033, Gibco Thermo Fisher), supplemented with 1% Glutamax(#35050061, Gibco Thermo Fisher), 2% B27, 1% N2, of 0.2% heparin (#07980 STEMCELL Technologies), 2-mercaptoethanol (50 mM), 1% Antibiotic-Antimycotic, Primocin (100 μg/mL)(#ant-pm-05 Invivogen, San Diego, CA), metronidazole (50 μg/mL) (M3761-5G, Sigma Aldrich) and the growth factors EGF (20 ng/mL) and human recombinant bFGF (20 ng/mL). Media supplemented with GDNF, RA and bFGF consisted of DMEM, supplemented with 2% B27, 1% N2, 2-mercaptoethanol (50 mM), 1% Antibiotic-Antimycotic, Primocin (100 μg/mL), metronidazole (50 μg/mL) and the growth factors human recombinant bFGF (20 ng/mL) (#78006, STEMCELL Technologies), all-trans retinoic acid (75 ng/mL) and human recombinant GDNF (50 ng/mL) (#78058, STEMCELL Technologies). Media was supplemented once weekly. Cell yield per mg of tissue was calculated by i) counting the number of cells after passage to calculate their

proliferation rate from original seeding densities, ii) multiplying the original total cell count after digestion by the proliferation rate and iii) dividing this value by the original tissue weight.

For human neurosphere immunocytochemistry, images were acquired from six randomly selected fields, covering a total area of 49.4 mm^2 per sample. The proportions of NGFR+, ITGA6+, and TUBB3+ cells were quantified using an automated Fiji (ImageJ) macro as follows: Rolling ball background subtraction was applied to reduce uneven illumination. Auto-local-thresholding (Phansalkar method) was applied to segment nuclei, followed by mask conversion and watershed transformation to separate closely associated nuclei in DAPI channel images. Cell counts were obtained using particle analysis. For the immunohistochemically labeled channels contrast was enhanced using Contrast Limited Adaptive Histogram Equalization (CLAHE) to improve visibility of structures and binary thresholding was performed using the MaxEntropy method to segment labeled structures and measurements were extracted via ROI analysis from the nuclei masks to count the number of overlapping labeled cells and nuclei and calculate the proportions of immunoreactive cells.

Statistical Analysis

All details of statistical analysis can be found in the figure legends. Data analysis was performed using GraphPad Prism v7 (GraphPad Software Inc., San Diego, USA). For all analyses $p < 0.05$ was considered significant. All data were presented as mean ± standard error of the mean (SEM), unless otherwise stated.

References

[1] Mallon BS, Shick HE, Kidd GJ, Macklin WB. Proteolipid promoter activity distinguishes two populations of NG2-positive cells throughout neonatal cortical development. Journal of Neuroscience 2002;22(3):876-85.

[2] Mueller JL, Leavitt AR, Rahman AA, Han CY, Ott LC, Mahdavian NS, Carbone SE, King SK, Burns AJ, Poole DP, Hotta R, Goldstein AM, Stavely R. Highly neurogenic glia from human and mouse myenteric ganglia generate functional neurons following culture and transplantation into the gut. Cell Reports 2024;43(11).

[3] Stavely R, Hotta R, Picard N, Rahman AA, Pan W, Bhave S, Omer M, Ho WLN, Guyer RA, Goldstein AM. Schwann cells in the subcutaneous adipose tissue have neurogenic potential and can be used for regenerative therapies. Science Translational Medicine 2022;14(646):eabl8753.

[4] Stavely R, Rahman AA, Mueller JL, Leavitt AR, Han CY, Pan W, Kaiser KN, Ott LC, Ohkura T, Guyer RA, Burns AJ, Koppes AN, Hotta R, Goldstein AM. Mature enteric neurons have the capacity to reinnervate the intestine with glial cells as their guide. Neuron 2024;112(18):3143-60.e6.

[5] Guyer RA, Stavely R, Robertson K, Bhave S, Mueller JL, Picard NM, Hotta R, Kaltschmidt JA, Goldstein AM. Single-cell multiome sequencing clarifies enteric glial diversity and identifies an intraganglionic population poised for neurogenesis. Cell Rep 2023;42(3):112194.

[6] Ott LC, Han CY, Mueller JL, Rahman AA, Hotta R, Goldstein AM, Stavely R. Bone Marrow Stem Cells Derived from Nerves Have Neurogenic Properties and Potential Utility for Regenerative Therapy. International Journal of Molecular Sciences 2023;24(6):5211.

[7] Mueller JL, Han C, Leavitt A, Chauhan V, Ott L, Guyer RA, Uesaka T, Enomoto H, Cheng L, Hotta R, Burns AJ, Stavely R, Goldstein AM. Intramuscular enteric glia persist in Hirschsprung disease and undergo neurogenesis in response to GDNF-NCAM1 signaling. Scientific Reports 2025;15(1):33200.

[8] Hotta R, Pan W, Bhave S, Nagy N, Stavely R, Ohkura T, Krishnan K, de Couto G, Myers R, Rodriguez-Borlado L, Burns AJ, Goldstein AM. Isolation, Expansion, and Endoscopic Delivery of Autologous Enteric Neuronal Stem Cells in Swine. Cell Transplantation 2023;32:09636897231215233.
