# Supplementary material for: Glial Cell Line-derived Neurotrophic Factor and Retinoic Acid Synergy Unlocks Neurogenesis in Adult Myenteric Glia/Neural Progenitors
Source: Cell Mol Gastroenterol Hepatol. 2026 Jan 5;20(5):101722. doi: 10.1016/j.jcmgh.2025.101722 (PMC12933833; doi:10.1016/j.jcmgh.2025.101722)
Supplement: Extended PDF [file mmc3.pdf]

cmgh
CELLULAR AND MOLECULAR GASTROENTEROLOGY AND HEPATOLOGY

RESEARCH LETTERS

Glial Cell Line-derived Neurotrophic Factor and Retinoic Acid Synergy Unlocks Neurogenesis in Adult Myenteric Glia/Neural Progenitors

The culture of myenteric enteric neural stem/progenitor cells (ENPs) is: (1) an important resource for understanding the biology of enteric neurons (ENs) and enteric glial cells (EGCs)[1–4]; and (2) a promising cell therapy for regenerating the enteric nervous system (ENS) in the gastrointestinal tract of patients with enteric neuropathies.[5,6] Despite this growing interest in modeling the ENS in vitro, current protocols remain suboptimal, with postnatal-derived cell cultures often dominated by EGCs and enteric mesenchymal cells (EMCs) at the expense of ENs.[7–9] Understanding the factors controlling culture heterogeneity and neurogenesis is essential for optimizing in vitro protocols and developing restorative cell therapies. Here, we investigated variables including donor age, culture adherence, and growth factors to understand and leverage their influence on the cellular composition of postnatal ENS cultures, with the aim of enhancing postnatal neurogenesis.

To determine the impact of donor age on the properties of ENS culture, neurospheres were cultured[1] from the muscularis propria of 0.5- and 12-month-old BAF53b::TdTomato (TdT); Plp1-GFP dual-reporter mice[2] to selectively label enteric glial/neural progenitor cells (EG/NP) (GFP fluorescence) and ENs (TdT fluorescence) (Figure 1*A*). *Sox10* (EG/NP) expression was unchanged between 0.5- and 12-month neurospheres, whereas *Elavl4* (EN) decreased and *Pdgfra* (EMC) increased with age (Figure 1*Ai*). In neurosphere image analysis, BAF53b-TdT fluorescence showed that neurogenesis was reduced by 2 months, with further decline at 12 months (Figure 1*Aii*, Supplementary Figure 1*A* and *B*). Postnatal EG/NPs acutely lose their neuronal differentiation potential similar to in vivo observations,[10] emphasizing age as a key experimental and translational variable for the production of ENs.

To test the influence of culture adherence conditions, adult-derived neurospheres were grown in free-floating 3D cultures and in adherent monolayers (Supplementary Figure 1*C* and *D*). *Plp1* and *Ngfr* levels were similar in both conditions, whereas cells were less differentiated in monolayers with reduced *Gfap, Phox2b,* and *Elavl4* (Supplementary Figure 1*E*). Neurospheres in 3D cultures yielded fewer singlets by flow cytometry after digestion than monolayers (26.7% ± 1.7% vs 32.3% ± 0.44%; $P < .01$), consistent with neurospheres having greater resistance to dissociation. Thus, monolayer cultures, which are more amenable to dissociation, yielded better recovery of ENs and EG/NP singlets than free-floating cultures (Supplementary Figure 1*F*). Monolayer cultures therefore offer advantages for protocols requiring cell suspensions, whereas 3D conditions improve cell differentiation (Supplementary Figure 2*A*).

To manipulate and optimize adult EG/NP proliferation and neurogenesis the ligands basic fibroblast growth factor (bFGF), retinoic acid (RA), and glial cell line-derived neurotrophic factor (GDNF), which play key roles in neural development, were tested individually and in combination (Supplementary Figure 3*A*). Positive control media (+CM), known to support neurosphere formation,[1] and basal control media (−CM) were included as comparators (Supplementary Figure 2*B*). bFGF expanded adult progenitors but did not increase numbers of ENs (Figure 1*B* and *Ci*). RA + GDNF synergistically increased neuronal differentiation 6.2-fold while moderately expanding EG/NPs (Figure 1*B* and *Ci*). To combine the proliferative and neurogenic properties of these growth factors, we utilized GRF media (GDNF + RA + bFGF) and compared it with +CM (Figure 1*D* and *Dii*, Supplementary Figure 3*B*). After 2 weeks, *Plp1, Gfap,* and *Ngfr* were comparable between groups, whereas *Pdgfra* was lower and *Phox2b/Elavl4* were higher in GRF (Figure 1*D*). Flow cytometry after monolayer expansion (Figure 1*Di*) showed GRF increased ENs 3.6-fold and EG/NPs 1.2-fold (Figure 1*Dii*), indicating enhanced neurogenesis without compromising progenitor maintenance. GRF did not significantly increase survival of sorted mature ENs as compared with +CM, indicating GRF enhances EN numbers via neurogenesis (Supplementary Figure 3*C–E*). In sorted Plp1+ EG/NPs, RA promoted the expression of *Ret* >12-fold, providing a mechanism for RA to enhance GDNF-driven neurogenesis via its receptor (Figure 1*E*). Purified EG/NPs in GRF media produced ENs, including representative intrinsic ENS subtypes nNOS, Calretinin, and Galanin (Supplementary Figure 4*A*; Figure 1*F*).

To translate our findings with mouse cells to human, we obtained human muscularis propria from patients aged 9 weeks to 49 years (Supplementary Figure 2*C*; Figure 2*A* and *B*), generated neurospheres, and cultured them in +CM or GRF. After 2 weeks, GRF neurospheres expressed increased *PLP1, NGFR,* and *PHOX2B* compared with +CM (Figure 2*C* and *D*). Similar to rodents, an age-related decline in *PHOX2B*, but not *PLP1*, was observed (Supplementary Figure 4*B*). Following monolayer expansion (Figure 2*E*), GRF yielded fewer cells (Figure 2*F*), but higher proportions of ITGA6+, NGFR+, and TUBB3+ cells by immunostaining (Figure 2*G–I*). Thus, like our observations with mouse cells, these data indicate that GRF media provides highly neurogenic human cell populations in vitro.

Our findings highlight that culture conditions can be tailored to dictate the numbers of glial and neuronal cells that can be generated in culture, depending on experimental or translational goals. bFGF robustly expanded adult EG/NPs but with limited production of neurons, while RA + GDNF increased neuronal

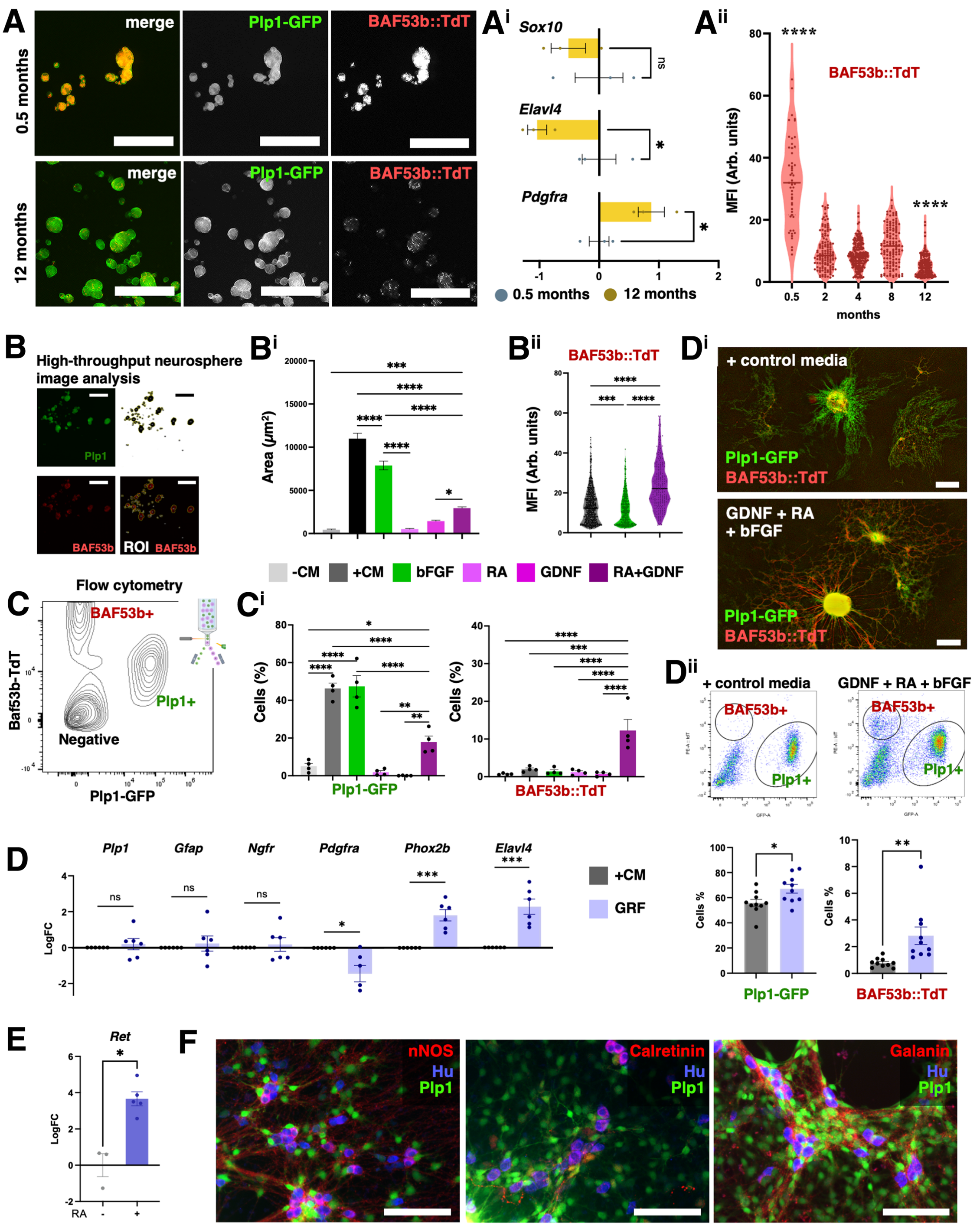

Figure 1. **(*A*) Neurospheres from 0.5- and 12-month-old BAF53b::TdT;Plp1-EGFP mice.** (*Ai*) PCR data from these neurospheres. n = 3 mice per group. (*Aii*) Quantification of MFI of TdT in GFP-binarized ROI. n = 51–224 neurospheres per group; individual data points from all wells combined for each age group. (*B*) Neurosphere analysis method of GFP signal binarization, region of interest (ROI) selection, and TdT mean fluorescence intensity (MFI) quantification. (*Bi*) Area (μm^2) per well covered by neurospheres in −CM, +CM, bFGF, GDNF, RA, and RA+GDNF media; n = 4 cultures. (*Bii*) TdT MFI in GFP-defined neurospheres. n = 248–639 neurospheres. (*C*) Flow cytometry gating examples. (*Ci*) Percentage of EG/NPs (Plp1-EGFP) and ENs (BAF53b-TdT). n = 4 cultures. (*D*) Quantitative PCR in neurospheres from the +CM and GRF media groups. n = 6 independent cultures per group. (*Di*) Representative images of monolayer cultures in +CM and GRF media. (*Dii*) Representative flow cytometry plots and quantification of EG/NPs and ENs in the +CM and GRF media groups. n = 10 independent cultures per group. (*E*) Expression of *Ret* in neurospheres cultured with and without RA. n = 3–5 cultures. (*F*) nNOS, Calretinin, and Galanin neurons in GRF media cultures. All data are mean ± SEM unless stated. Unpaired *t*-test (*Ai, Di, Dii, E*), Kruskal-Wallis ANOVA with Dunn's posthoc test (*Aii*), One-way ANOVA with Holm-Sidak (*Bi, Bii, Ci*), *$P < .05$; **$P < .01$; ***$P < .001$; ****$P < .0001$. Scale bars = 1000 um (*A*), 500 um (*D*), 100 um (*F*).

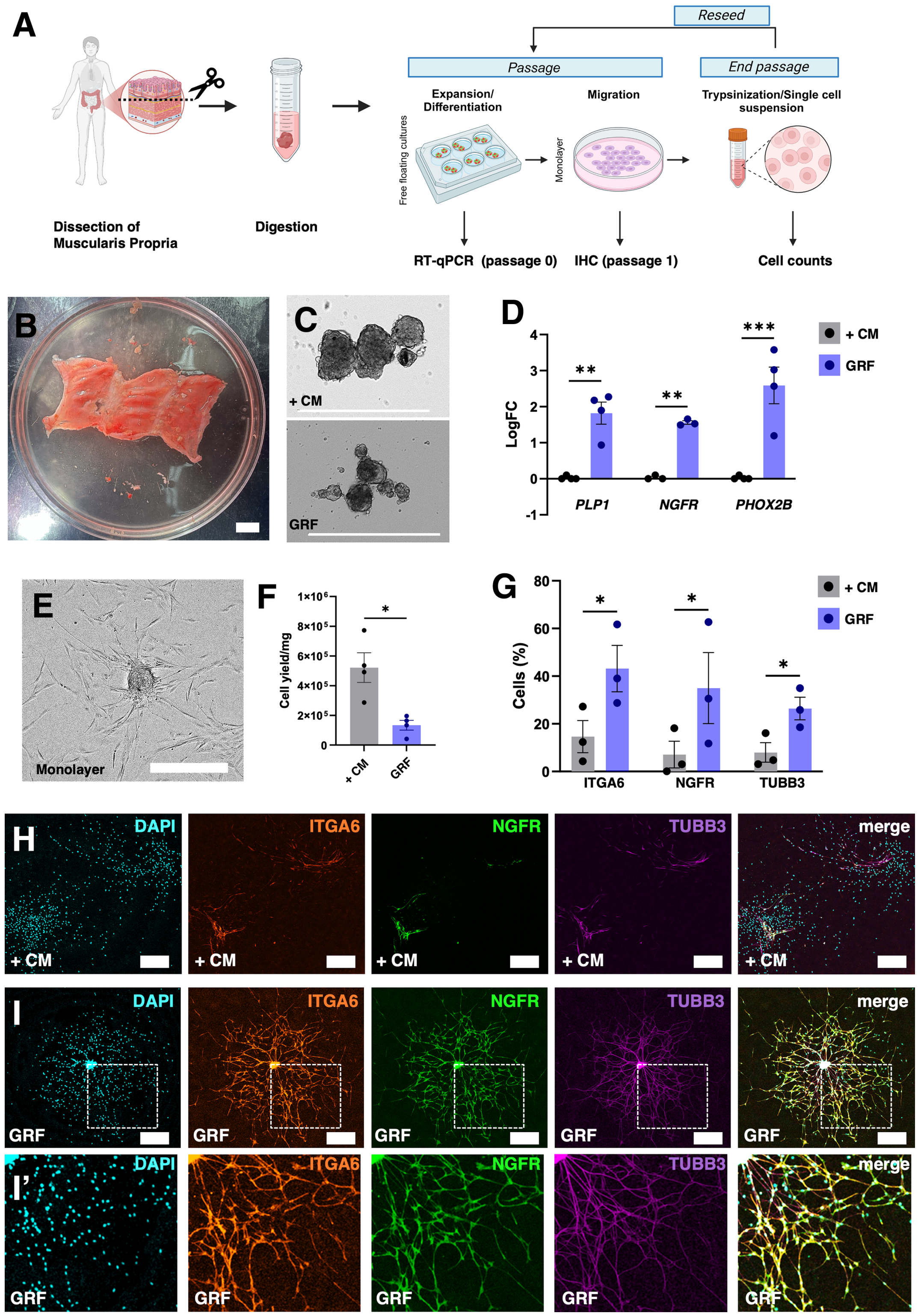

Figure 2. ***(A)* Schematic of human intestinal neurospheres cultured in +CM or GRF media.** (*B*) Representative muscularis propria tissue after dissection. (*C*) Representative neurospheres in +CM and GRF media. (*D*) EG/NP markers *PLP1, NGFR*, and neuronal marker *PHOX2B* after 10–14 days free-floating culture; n = 3–4 subjects. (*E*) Neurospheres transferred to monolayer in GRF media. (*F*) Cell yield per mg of tissue; n = 4. (*G*) Percentage of ITGA6+, NGFR+, and TUBB3+ cells; n = 3; 2-way ANOVA. (*H* and *I*) Representative immunohistochemistry images for ITGA6, NGFR, and TUBB3 in +CM (*H*) and GRF (*I*). All data are mean ± SEM. Two-way ANOVA with Holm-Sidak (*D* and *G*), ratio paired *t*-test (*F*). $^{*}P < .05$; $^{**}P < .01$; $^{***}P < .001$. Scale bars: 1 cm (*B*), 500 μm (*C, E, H* and *I-I'*).

differentiation, thereby demonstrating the ability to tune cell fate. Our results not only align with reports of age-related decline in ENS neurogenesis in vivo,[10] but importantly, also demonstrate that adult EG/NPs retain neurogenic capability when appropriately stimulated in vitro. Insights from iPSC-derived systems could further refine approaches to culture adult progenitors, and future studies should assess potential sex-dependent effects. Optimizing culture conditions and exposure to pro-neurogenic cues represents a critical step towards the establishment of reproducible in vitro models of the ENS and the development of adult neural cell therapies, where precise manipulation of EG/NPs and ENs is essential.

CHRISTOPHER Y. HAN
VIPIN CHAUHAN
JESSICA L. MUELLER
AKI KASHIWAGI
ALAN J. BURNS
RHIAN STAVELY
Department of Pediatric Surgery
Massachusetts General Hospital
Harvard Medical School
Boston, Massachusetts

Supplementary Material

Note: To access the supplementary material accompanying this article, visit the full text version at https://doi.org/10.1016/j.jcmgh.2025.101722.

References

1. Guyer RA, et al. Cell Rep 2023;42:112194.
2. Stavely R, et al. Neuron 2024;112:3143–3160.e6.
3. Schneider L, et al. Front Immunol 2024;15:1401751.
4. Gomes P, et al. Neurogastroenterol Motil 2009;21:870-e62.
5. Rahman AA, et al. JCI Insight 2024;9:e179755.
6. Burns AJ, et al. Dev Biol 2016;417:229–251.
7. Binder E, et al. PLoS One 2015;10:e0119467.
8. Mueller JL, et al. Cell Rep 2024;43:114919.
9. Schonkeren SL, et al. Neurogastroenterol Motil 2022;34:e14215.
10. Pham TD, et al. J Comp Neurol 1991;314:789–798.

Most current article

© 2026 The Authors. Published by Elsevier Inc. on behalf of the AGA Institute. This is an open access article under the CC BY license (http://creativecommons.org/licenses/by/4.0/).
2352-345X
https://doi.org/10.1016/j.jcmgh.2025.101722

Received June 18, 2025. Accepted December 30, 2025.

Correspondence
Address correspondence to: Rhian Stavely, PhD, Massachusetts General Hospital, Harvard Medical School, Department of Pediatric Surgery, 185 Cambridge Street, CPZN, 6100, Boston, Massachusetts 02114. e-mail: rstavely@mgh.harvard.edu.

Acknowledgments
The Department of Pediatric Surgery at Massachusetts General Hospital includes: Abigail R. Leavitt, Harsh Panchal, Takahiro Ohkura, Leah C. Ott, Ahmed A. Rahman, and Ryo Hotta from the Department of Pediatric Surgery, Massachusetts General Hospital, Harvard Medical School, Boston, Massachusetts.

Conflicts of interest
These authors disclose the following: Rhian Stavely and Ryo Hotta are inventors on United States provisional patent application 63/ 659,137 submitted by The General Hospital Corporation that covers "Isolation of enteric neurons and progenitors from the enteric ganglia for cell therapy". The remaining authors disclose no conflicts.

Funding
This work was supported by National Institutes of Health grants R21HD106036 and Charles H. Hood Foundation Child Health Research Award to Rhian Stavely.

Cellular and Molecular Gastroenterology and Hepatology, Volume *20*

Supplemental information

Glial Cell Line-derived Neurotrophic Factor and Retinoic Acid Synergy Unlocks Neurogenesis in Adult Myenteric Glia/Neural Progenitors

Christopher Y. Han, Vipin Chauhan, Jessica L. Mueller, Aki Kashiwagi, Alan J. Burns, and Rhian Stavely

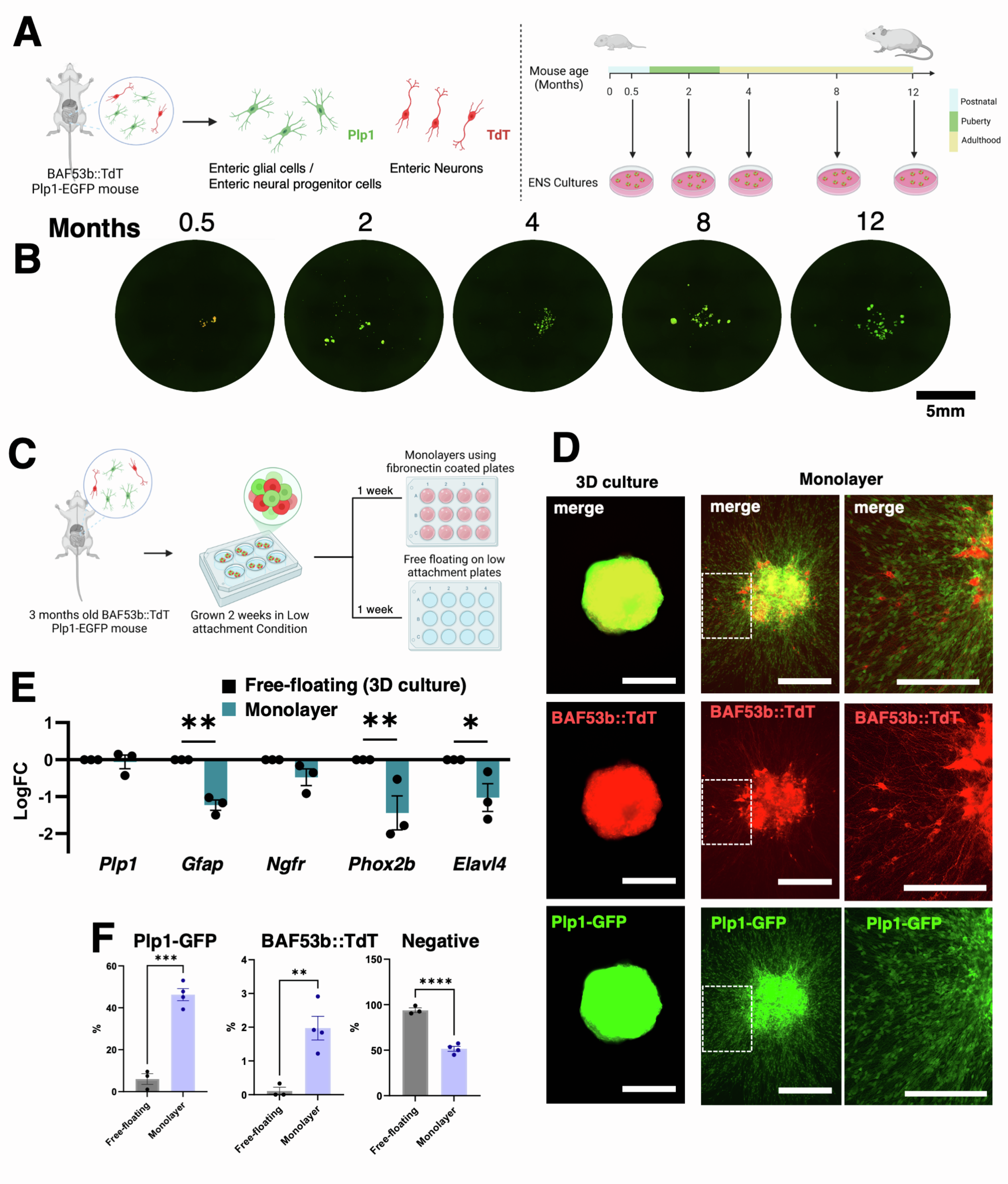

Supplementary Figure 1. A) Neurogenesis declines in enteric neurospheres with increasing age of mice. Schematic overview of neurosphere cultures from BAF53b::TdT; Plp1-EGFP dual-reporter mice at a range of ages. **B)** Cells isolated from BAF53b::TdT; Plp1-EGFP dual-reporter mice at 2 weeks, 2 months, 4 months, 8 months, and 1 year of age give rise to GFP+ and TdT+ neurospheres in culture, with Plp1-EGFP serving as a EG/NP marker and BAF53b::TdT as a neuronal marker. Images are representative of whole wells in culture plates. Scale bar = 5mm. **C)**

Culture format influences cell composition and assay sensitivity in enteric neural cultures. Schematic representation of neurosphere cultures grown under monolayer and free-floating conditions (3D culture) from BAF53b::TdT; Plp1-EGFP mice. **D)** Representative images of neurospheres generated from BAF53b::TdT; Plp1-EGFP dual-reporter mice, in the free-floating group at the 3-week timepoint of culture and the monolayer group at the 3-week timepoint of culture. Scale bars = 500um and 250um for right insets. **E)** Quantitative PCR of *Plp1, Gfap, Ngfr, Phox2b*, and *Elavl4* in neurospheres from the free-floating and monolayer groups. Data are shown as mean ± SEM. Two-way ANOVA with Holm-Sidak posthoc test, $^{*}p < 0.05$, $^{**}p < 0.01$; n = 3 mice per group. **F)** Quantification of flow cytometry for EG/NPs (Plp1-EGFP), ENs (BAF53b::TdT), and EMCs (double-negative cells) from free-floating and monolayer groups. Data are shown as mean ± SEM. One-way ANOVA with Holm-Sidak posthoc test, $^{**}p < 0.01$, $^{***}p < 0.001$, $^{****}p < 0.0001$; n = 3-4 wells per group.

A) Comparison between monolayer and neurosphere culture systems

Culture format	Benefits	Assays
**Free-floating neurospheres** (3D culture)	- Better mimics *in vivo* 3D structure<br>- Higher rates of neuronal differentiation<br>- More scope for expansion	- PCR<br>- Transgenic fluorescence<br>- Neurosphere coverage
**Monolayer**	- Easier to maintain and passage<br>- More amenable to generation of single-cell suspensions for controlled passaging and assays	- Immunocytochemistry<br>- Neurite outgrowth<br>- Cell yield<br>- Flow cytometry<br>- PCR

B) Media definitions

Media	+CM	-CM	FGF	RA	GDNF	GR	GRF
**DMEM**	+	+	+	+	+	+	+
**NeuroCult**	+	-	-	-	-	-	-
**B27**	+	+	+	+	+	+	+
**N2**	+	+	+	+	+	+	+
**Anti-Anti**	+	+	+	+	+	+	+
**2-ME**	+	-	-	-	-	-	-
**IGF**	+	-	-	-	-	-	-
**bFGF**	+	-	+	-	-	-	+
**RA**	+	-	-	+	-	+	+
**GDNF**	-	-	-	-	+	+	+

+CM, positive control media; -CM, negative control media; bFGF, fibroblast growth factor; RA, retinoic acid; GDNF, glial cell line-derived neurotrophic factor; GR, GDNF+RA; GFR, GDNF+RA+FGF; 2-ME, 2-mercaptoethanol; IGF, insulin-like growth factor, bFGF; Basic fibroblast growth factor.

C) Human sample details

ID	Age	Sex	Procedure	Tissue
**1**	9-week-old	M	Resection for Meckel's diverticulum	Ileum
**2**	6.5-month-old	M	Colostomy Closure for anorectal malformation	Sigmoid Colon
**3**	36-year-old	F	Right colectomy for colonic tumor	Transverse Colon
**4**	49-year-old	F	Colostomy closure following prior diversion for colorectal cancer	Transverse Colon

Supplementary Figure 2. A) Comparison of culture formats used in this study, outlining key benefits and downstream applications. Free-floating neurospheres (3D culture) better recapitulate *in vivo* three-dimensional architecture, exhibit higher rates of neuronal differentiation, and allow greater capacity for cellular expansion. Neurosphere cultures were used for gene expression analysis by PCR, assessment of transgenic reporter fluorescence, and quantification of neurosphere formation and coverage. Monolayer cultures are easier to maintain and passage and are more amenable to generation of single-cell suspensions for controlled passaging and downstream assays. Monolayer systems can be used for immunocytochemistry, neurite outgrowth analysis, cell yield quantification, flow cytometry, and PCR. **B)** Composition of culture media used across experimental conditions. Media components are indicated as present (+) or absent (−). +CM denotes positive control media and −CM denotes negative control media. Abbreviations: DMEM, Dulbecco's Modified Eagle Medium; bFGF/FGF, basic fibroblast growth factor; RA, retinoic acid; GDNF, glial cell line–derived neurotrophic factor; GR, GDNF + RA; GRF, GDNF + RA + FGF; B27 and N2, neuronal supplements; Anti-Anti, antibiotic–antimycotic; 2-ME, 2-mercaptoethanol; IGF, insulin-like growth factor. **C)** Clinical and demographic details of human intestinal samples used for primary cell isolation, including age, sex, surgical indication, and tissue source.

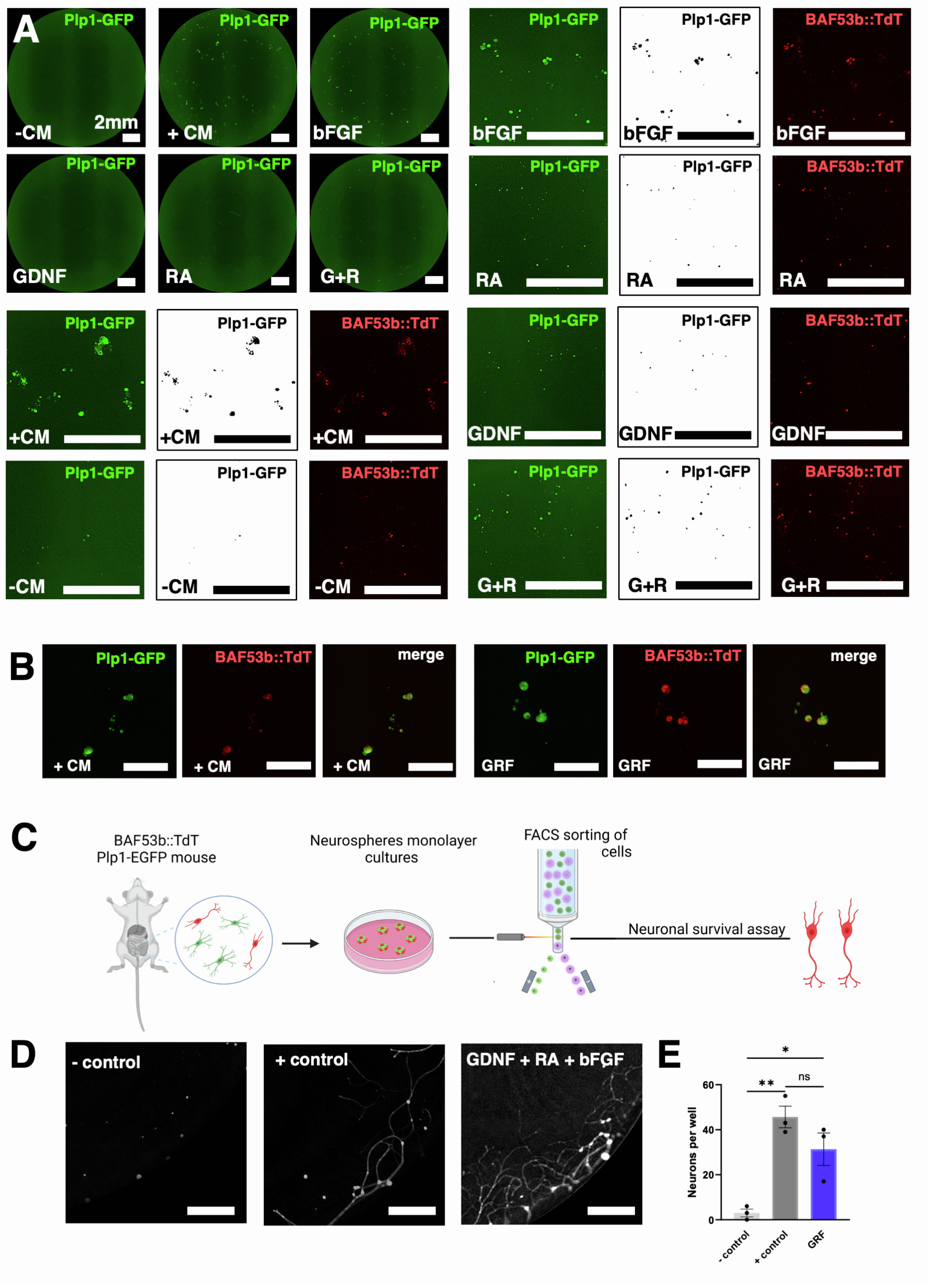
A
Plp1-GFP
BAF53b::TdT
-CM
2mm
+ CM
bFGF
GDNF
RA
G+R
+CM
B
merge
GRF
C
BAF53b::TdT
Plp1-EGFP mouse
Neurospheres monolayer cultures
FACS sorting of cells
Neuronal survival assay
D
- control
+ control
GDNF + RA + bFGF
E
Neurons per well
60
40
20
0
- control
+ control
GRF
*
**
ns

Supplementary Figure 3. A) Representative GFP-channel images of neurospheres generated from 3-month-old BAF53b::TdT; Plp1-EGFP dual-reporter mice and cultured in negative control media (-CM), positive control media (+CM), bFGF media, RA, GDNF or GDNF + RA (G+R) media conditions. Representation of GFP fluorescence binarization and TdT fluorescence for neurospheres. Scale bars = 2mm. **B)** GDNF, RA and bFGF (GRF) improves enteric neuronal composition while maintaining enteric glial/neural progenitor expansion in adult-derived neurosphere cultures. Representative images of neurospheres generated from BAF53b::TdT; Plp1-EGFP dual-reporter mice, cultured in the +CM media and GDNF + RA + bFGF (GRF) media. **C)** GDNF, RA and bFGF (GRF) improves enteric neuronal composition while maintaining enteric glial/neural progenitor expansion in adult-derived neurosphere cultures. Schematic representation of neurosphere cultures grown in positive control media (+CM), bFGF, and GDNF + RA + bFGF (GRF) media. **B)** Representative images of neurospheres generated from BAF53b::TdT; Plp1-EGFP dual-reporter mice, cultured in the +CM media or GDNF + RA + bFGF (GRF) media. Scale bars = 2mm. **C)** Schematic illustration of ENs (BAF53b::TdT) sorted from BAF53b::TdT; Plp1-EGFP dual-reporter mice, used for EN survival assays. **D)** Representative TdT-channel images of ENs (BAF53b::TdT) sorted from BAF53b::TdT; Plp1-EGFP dual-reporter mice, then cultured in negative control (-CM), positive control (+CM), or GDNF + RA + bFGF (GRF) media conditions, at the 2-week timepoint of culture. Scale bars = 500um. **E)** Quantification of neurons per well in ENs (BAF53b::TdT) as above. Data are shown as mean ± SEM. One-way ANOVA with Holm-Sidak posthoc test, $^{*}p < 0.05$, $^{**}p < 0.01$; $n = 3$ independent cultures per group.

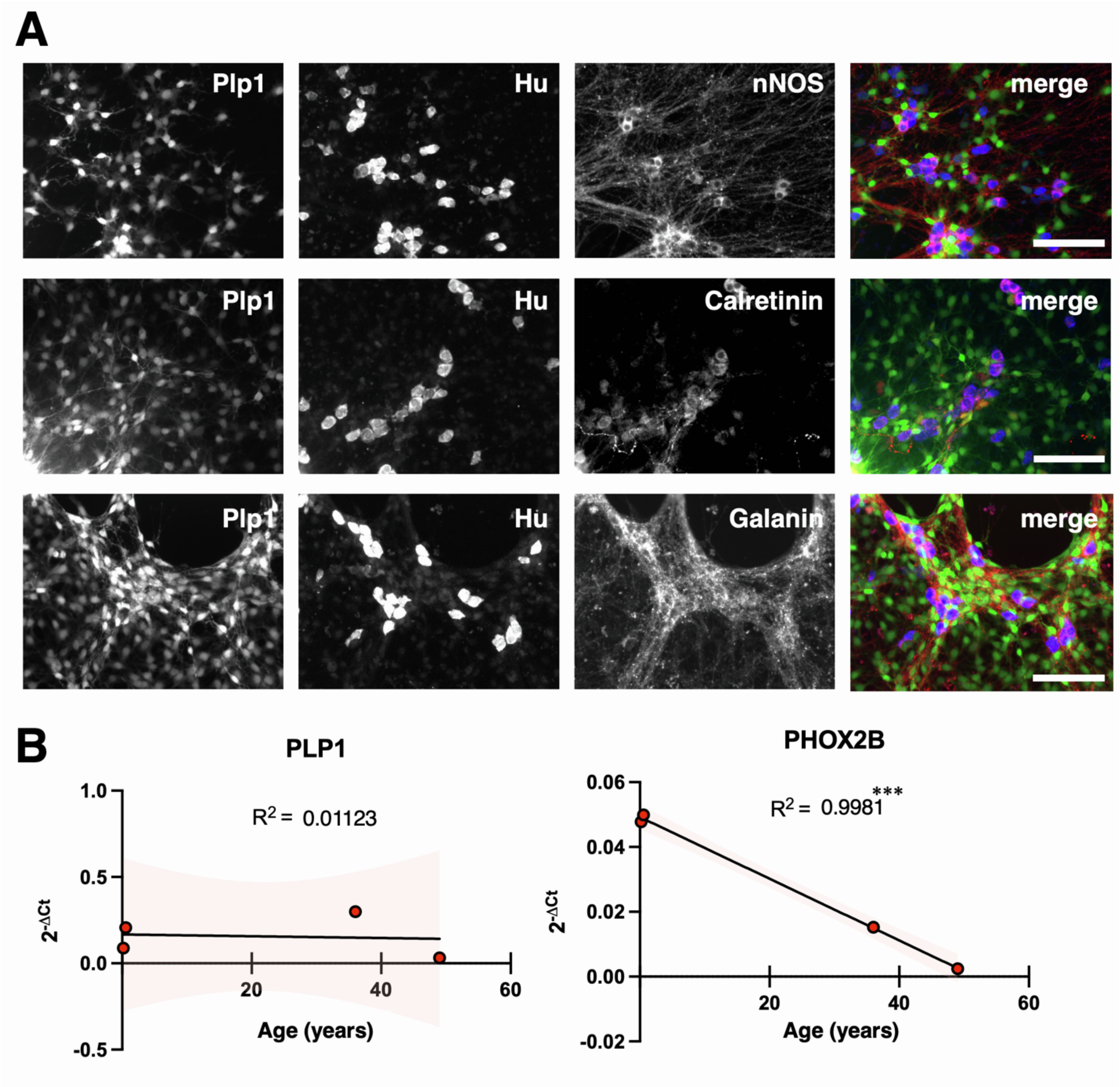

Supplementary Figure 4. A) Enteric glial/neural progenitor cells (EG/NPs) sorted for Plp1-GFP expression and cultured in GDNF + RA + bFGF (GRF) media undergo neurogenesis and give rise to nNOS, Calretinin and Galanin immunoreactive neurons in culture. Scale bars = 100 um. **B)** Linear regression analysis of the expression of PLP1 or PHOX2B ($2^{-\Delta Ct}$) against the age (years) of human subjects from which cultures were derived. Data are from samples grown in GRF media. Data shows individual points (red circles), regression line (black line) and 95% confidence intervals (shaded region). Significance of the slope, $^{***}p < 0.001$.

Materials/Methods

All authors had access to the study data and had reviewed and approved the final manuscript. All data and analytic methods are provided in the manuscript. All raw data and study materials will be made available to other researchers upon reasonable request.

Animals

Animal experimentation was performed according to experimental protocols approved by the Institutional Animal Care and Use Committees IACUC (2009N000239) of Massachusetts General Hospital. Plp1-EGFP mice were gifted to the Goldstein laboratory by Wendy Macklin [1]. BAF53b::Cre (stock number 027826) and (R)26-tdTomato (stock number 007914) mice were purchased from The Jackson Laboratory. BAF53b::tdT; Plp1-EGFP dual glia and neuron reporter mice were generated as previously described [2-4]. All mice were housed and bred under specific-pathogen-free conditions at the Center for Comparative Medicine animal facility at Massachusetts General Hospital (MGH). Rodents were housed in Allentown Inc. rectangular caging (160 cages per individually ventilated cage racks; which uses blower at 60 air changes per hour) under a 12h:12h light:dark cycle from 7 am – 7 pm. Bedding consisted of Hardwood Sanichip; with Carefresh nesting material and mice had access to Prolab Isopro RMH 3000 chow mix (ScottPharma) *ad libitum*.

Generation of Heterogenous Mouse Cultures and Study Designs

Mice were euthanized and the small intestine was removed from duodenum to terminal ileum. The smooth muscle-myenteric plexus (SMMP) layer was dissected from underlying tissue in PBS and digested for 35 minutes at 37 °C in dispase (250 µg/mL; #07923 STEMCELL Technologies,

Vancouver, BC) and collagenase XI (#C7657-25MG 1 mg/mL; Sigma–Aldrich, Darmstadt, Germany). Following digestion, the cells were filtered through a 40-μm filter and plated in their respective media formulation **(Supplementary Figure 2B)**. Positive control media (+CM) contained a 1:1 mixture of DMEM (+ Glucose + Glutamine, Thermo Fisher, Waltham, MA) and NeuroCult Basal Media (#05700, STEMCELL Technologies,) supplemented with 20 ng/mL bFGF (STEMCELL Technologies, #78003), 20 ng/mL IGF-I (#RP-10931 Thermo Fisher, Waltham, MA)1, 2% B-27 supplement (#, 12587001, Gibco Thermo Fisher), 1% N-2 supplement (#17502001, Gibco Thermo Fisher), 50 mM 2-mercaptoethanol(#21985023, Thermo Fisher), 75 ng/mL all-trans retinoic acid (#R2625-100MG, Sigma-Aldrich) and 1% Antibiotic-Antimycotic (#15240062, Gibco Thermo Fisher) as previously reported [5]. Negative control media (-CM) contained only 2% B27 supplement, 1% N_2 supplement and 1% Antibiotic-Antimycotic in DMEM (#11995073, Gibco Thermo Fisher) and was supplemented with different combinations of bFGF (20 ng/mL), all-trans retinoic acid (75 ng/mL) and/or GDNF (50ng/mL) (#10788-108, FUJIFILM Irvine Scientific, Santa Ana, CA). All experiments were conducted using a standard humidified cell culture incubator (37˚C, 5% CO_2 and atmospheric O_2) with media supplemented once weekly.

High throughput image-based analysis of BAF53b::tdT; Plp1-EGFP neurospheres.

For experiments examining the effects of age and media compositions on heterogenous cell cultures, BAF53b::tdT; Plp1-EGFP mice were sacrificed and single cell suspensions were generated as above. Cells were plated at a density of 50,000 cells per well in 24-well ultra-low attachment plates (Corning Costar® 24-well Clear Flat Bottom Ultra-Low Attachment Multiple Well Plates Cat# 3473). After two weeks, whole-plate tile scans were acquired using a Keyence BZX-700 All-In-One Microscopy System (Keyence America, Itasca). Image processing was

performed in ImageJ (NIH, Bethesda), where individual wells were isolated and analyzed as follows: The Plp1-EGFP channel was binarized using auto-thresholding with the MaxEntropy algorithm, followed by despeckling to remove outliers smaller than 2 µm. Neurospheres were segmented using the Watershed function, and the "Analyze Particles" command was used to quantify and measure the area occupied by Plp1-EGFP+ neurospheres, filtering for objects >20 µm. Regions of interest (ROIs) for each neurosphere generated from the particle analysis were utilized to measure the mean grey value (mean fluorescence intensity, MFI) of BAF53b::tdT expression within each neurosphere.

RNA Isolation / qPCR

RNA isolation and RT-qPCR was performed as previously described [2, 6] via a RNeasy Mini kit (#74106, Qiagen, Hilden, Germany) as of manufacturer's instructions. Primer sequences for gene amplification in mouse and human samples are:

Gene	Forward sequence (5'→3')	Reverse sequence (5'→3')
**Gapdh (Ms)**	AGG TCG GTG TGA ACG GAT TTG	TGT AGA CCA TGT AGT TGA GGT CA
**Plp1 (Ms)**	TGA GCG CAA CGG TAA CAG G	GGG AGA ACA CCA TAC ATT CTG G
**Gfap (Ms)**	GGG GCA AAA GCA CCA AAG AAG	GGG ACA ACT TGT ATT GTG AGC C
**Ngfr (Ms)**	CCT GGA CAG TGT TAC GTT CTC	ACA CAG GGA GCG GAC ATA CT
**Phox2b (Ms)**	GGG CTA AGT TTC GCA AGC AG	CAG TGC TGT CGG GAT CAG TG
**Elavl4 (Ms)**	GCC TCA GGT GTC AAA TGG ACC	ACC CTA AAC TCT GTC CTG TGA T
**Pdgfra (Ms)**	ATG AGA GTG AGA TCG AAG GCA	CGG CAA GGT ATG ATG GCA GAG
**Sox10 (Ms)**	CGG ACG ATG ACA AGT TCC CC	GTG AGG GTA CTG GTC GGC T
**Ret (Ms)**	GCA TGT CAG ACC CGA ACT CC	CGC TGA GGG TGA AAC CAT CC
**GAPDH (Hu)**	GGA GCG AGA TCC CTC CAA AAT	GGC TGT TGT CAT ACT TCT CAT GG
**PLP1 (Hu)**	TGC TGA TGC CAG AAT GTA TGG	GCA GAT GGA CAG AAG GTT GGA
**NGFR (Hu)**	CCG TTG GAT TAC ACG GTC CAC	TGA AGG CTA TGT AGG CCA CAA
**TUBB3 (Hu)**	GGC CAA GGG TCA CTA CAC G	GCA GTC GCA GTT TTC ACA CTC

Quantification cycle (Ct) values were normalized to *Gapdh/GAPDH* expression within each sample as an internal control, and the Log2 fold change (FC) was calculated between

corresponding sample conditions from the same mouse or subject. All reactions were performed in duplicate.

Flow cytometry analysis

Analysis by flow cytometry was conducted on samples generated from BAF53b::tdT; Plp1-EGFP mice. To generate single cell suspensions, free-floating neurospheres were dissociated for 45 minutes using Accutase (STEMCELL Technologies), or monolayer cultures on fibronectin-coated plates (sigma, #F1141, 1:100 for 1h) were dissociated for 10 minutes into a single-cell suspensions using .025% Trypsin-EDTA (#25200056, Gibco Thermo Fisher) at 37C°C. Samples were filtered through a 35-μm cell strainer (#6475025, Electron Microscopy Sciences, Hatfield, PA) and stained with DAPI(#D1306, Invitrogen Thermo Fisher) serving as a viability marker. Cell sorting was performed with BD FACSAria cell sorter (BD Biosciences, Franklin Lakes, New Jersey) instruments. Flow cytometric analysis was conducted using FlowJo software (FlowJo, LLC, OR).

Neuronal survival assay

Cells were isolated from the small intestine of BAF53b::tdT; Plp1-EGFP mice as described above. ENs were isolated using fluorescence activated cell sorting (FACS) as previously described [4]. Briefly, the heterogenous population of cells was plated at a density of 5×10^4 cells/cm^2 on a fibronectin-coated (sigma, #F1141, 1:100 for 1h) 24-well cell culture plate cultured for 2 days in media comprised of DMEM/F12 media (ThermoFisher, Gibco) containing 10% FBS (#10438-018, ThermoFisher, Gibco) and 1% penicillin-streptomycin (ThermoFisher, Gibco, 15140122). Monolayers were trypinized as above and FACS was conducted using a BD FACSAria cell sorter (BD Biosciences) to collect GFP+ EG/NPs and tdT+ ENs.. For neuronal survival assays

BAF53b::tdT+ ENs were cultured in their respective media on 96-well fibronectin-coated flat-bottom plate at a density of 500 neurons per well. After two weeks in culture images of the entire wells were taken on a Keyence BZX-700 All-In-One Microscopy System and the number of neurons were quantified per well manually with ImageJ. Media was supplemented once weekly.

Immunocytochemistry

Monolayer cultures were fixed and immunolabeled as previously described [2, 7]. The following primary antibodies were utilized: rabbit anti-nNOS(C7D7) (4231, 1:200, Cell Signaling technology, Danvers, MA); rabbit anti-calretinin, (18-0211, 1:200, Invitrogen, Carlsbad, CA) rabbit anti-galanin (T4333, 1:2000, BMA Biomedicals, Augst, Switzerland), donkey anti-rabbit 647 (Invitrogen), mouse anti-tubulin β3 (801210, 1:400, conjugated to Alexa Fluor 647, BioLegend, San Diego, CA); mouse anti-CD271 (NGFR) (345104, 1:200, conjugated to FITC, Biolegend); rat anti-CD49f/ITGA6 (313616, 1:200; conjugated to APC, Biolegend). Cell nuclei were stained with DAPI (Invitrogen).

Human Gut Tissues

Colonic or ileal samples resected as part of required patient care at Massachusetts General Hospital were collected from subjects between 2 months and 49 years old **(Table 3)**. Tissues were stored overnight at 4°C in sterile PBS. The muscularis propria was mechanically separated from the mucosal, submucosal, and serosal layers using fine forceps and microdissection scissors under a stereoscopic microscope. For enzymatic dissociation, tissues were minced into approximately 5 mm fragments using sterile microdissection scissors and digested in a prewarmed enzymatic solution consisting of collagenase type XI (1 mg/mL) and dispase (0.6 U/mL) in DMEM/F12. The

digestion was carried out at 37°C for 4 h in a Mini Incubated Shaker (#76407-108, VWR International, Portland, OR) with intermittent trituration using glass serological pipettes of progressively smaller bore diameters (3 mm, 2 mm, and 1 mm) until the sample became liquefied. Large undigested tissue fragments were removed using a 1000 µm cell strainer (#4355100003 pluriStrainer, pluriSelect USA, El Cajon, USA), and single cells were obtained by passing the suspension through a 70 µm strainer (#22-363-548 Fisher Scientific, Pittsburgh, PA). Red blood cells were lysed by incubating the cell suspension in ACK Lysing Buffer (#A1049201, Gibco, ThermoFisher Scientific) for 7 min at room temperature.

Following isolation, cells were divided into equal aliquots and plated in their respective culture media at a density of 5–20 × 10^3 cells/cm^2 in ultra-low attachment plates for direct comparisons. As a positive control media (+CM) we utilized a defined formulation [8] which consisted of DMEM/F12(#11320033, Gibco Thermo Fisher), supplemented with 1% Glutamax(#35050061, Gibco Thermo Fisher), 2% B27, 1% N2, of 0.2% heparin (#07980 STEMCELL Technologies), 2-mercaptoethanol (50 mM), 1% Antibiotic-Antimycotic, Primocin (100 µg/mL)(#ant-pm-05 Invivogen, San Diego, CA), metronidazole (50 µg/mL) (M3761-5G, Sigma Aldrich) and the growth factors EGF (20 ng/mL) and human recombinant bFGF (20 ng/mL). Media supplemented with GDNF, RA and bFGF consisted of DMEM, supplemented with 2% B27, 1% N2, 2-mercaptoethanol (50 mM), 1% Antibiotic-Antimycotic, Primocin (100 µg/mL), metronidazole (50 µg/mL) and the growth factors human recombinant bFGF (20 ng/mL) (#78006, STEMCELL Technologies), all-trans retinoic acid (75 ng/mL) and human recombinant GDNF (50 ng/mL) (#78058, STEMCELL Technologies). Media was supplemented once weekly. Cell yield per mg of tissue was calculated by i) counting the number of cells after passage to calculate their

proliferation rate from original seeding densities, ii) multiplying the original total cell count after digestion by the proliferation rate and iii) dividing this value by the original tissue weight.

For human neurosphere immunocytochemistry, images were acquired from six randomly selected fields, covering a total area of 49.4 mm^2 per sample. The proportions of NGFR+, ITGA6+, and TUBB3+ cells were quantified using an automated Fiji (ImageJ) macro as follows: Rolling ball background subtraction was applied to reduce uneven illumination. Auto-local-thresholding (Phansalkar method) was applied to segment nuclei, followed by mask conversion and watershed transformation to separate closely associated nuclei in DAPI channel images. Cell counts were obtained using particle analysis. For the immunohistochemically labeled channels contrast was enhanced using Contrast Limited Adaptive Histogram Equalization (CLAHE) to improve visibility of structures and binary thresholding was performed using the MaxEntropy method to segment labeled structures and measurements were extracted via ROI analysis from the nuclei masks to count the number of overlapping labeled cells and nuclei and calculate the proportions of immunoreactive cells.

Statistical Analysis

All details of statistical analysis can be found in the figure legends. Data analysis was performed using GraphPad Prism v7 (GraphPad Software Inc., San Diego, USA). For all analyses $p < 0.05$ was considered significant. All data were presented as mean ± standard error of the mean (SEM), unless otherwise stated.

References

[1] Mallon BS, Shick HE, Kidd GJ, Macklin WB. Proteolipid promoter activity distinguishes two populations of NG2-positive cells throughout neonatal cortical development. Journal of Neuroscience 2002;22(3):876-85.

[2] Mueller JL, Leavitt AR, Rahman AA, Han CY, Ott LC, Mahdavian NS, Carbone SE, King SK, Burns AJ, Poole DP, Hotta R, Goldstein AM, Stavely R. Highly neurogenic glia from human and mouse myenteric ganglia generate functional neurons following culture and transplantation into the gut. Cell Reports 2024;43(11).

[3] Stavely R, Hotta R, Picard N, Rahman AA, Pan W, Bhave S, Omer M, Ho WLN, Guyer RA, Goldstein AM. Schwann cells in the subcutaneous adipose tissue have neurogenic potential and can be used for regenerative therapies. Science Translational Medicine 2022;14(646):eabl8753.

[4] Stavely R, Rahman AA, Mueller JL, Leavitt AR, Han CY, Pan W, Kaiser KN, Ott LC, Ohkura T, Guyer RA, Burns AJ, Koppes AN, Hotta R, Goldstein AM. Mature enteric neurons have the capacity to reinnervate the intestine with glial cells as their guide. Neuron 2024;112(18):3143-60.e6.

[5] Guyer RA, Stavely R, Robertson K, Bhave S, Mueller JL, Picard NM, Hotta R, Kaltschmidt JA, Goldstein AM. Single-cell multiome sequencing clarifies enteric glial diversity and identifies an intraganglionic population poised for neurogenesis. Cell Rep 2023;42(3):112194.

[6] Ott LC, Han CY, Mueller JL, Rahman AA, Hotta R, Goldstein AM, Stavely R. Bone Marrow Stem Cells Derived from Nerves Have Neurogenic Properties and Potential Utility for Regenerative Therapy. International Journal of Molecular Sciences 2023;24(6):5211.

[7] Mueller JL, Han C, Leavitt A, Chauhan V, Ott L, Guyer RA, Uesaka T, Enomoto H, Cheng L, Hotta R, Burns AJ, Stavely R, Goldstein AM. Intramuscular enteric glia persist in Hirschsprung disease and undergo neurogenesis in response to GDNF-NCAM1 signaling. Scientific Reports 2025;15(1):33200.

[8] Hotta R, Pan W, Bhave S, Nagy N, Stavely R, Ohkura T, Krishnan K, de Couto G, Myers R, Rodriguez-Borlado L, Burns AJ, Goldstein AM. Isolation, Expansion, and Endoscopic Delivery of Autologous Enteric Neuronal Stem Cells in Swine. Cell Transplantation 2023;32:09636897231215233.
